# Effects of *Eimeria maxima* infection doses on growth performance and gut health in dual-infection model of necrotic enteritis in broiler chickens

**DOI:** 10.3389/fphys.2023.1269398

**Published:** 2023-09-20

**Authors:** Doyun Goo, Janghan Choi, Hanseo Ko, Venkata Sesha Reddy Choppa, Guanchen Liu, Hyun Soon Lillehoj, Woo Kyun Kim

**Affiliations:** ^1^ Department of Poultry Science, University of Georgia, Athens, GA, United States; ^2^ Animal Bioscience and Biotechnology Laboratory, Beltsville Agricultural Research Center, Agricultural Research Service, United States Department of Agriculture, Beltsville, MD, United States

**Keywords:** broiler chicken, *Clostridium perfringens*, *Eimeria maxima*, intestinal health, necrotic enteritis

## Abstract

The objective of this study was to investigate the effects of the different doses of *Eimeria maxima* (*EM*) oocysts on growth performance and intestinal health in broiler chickens challenged with a dual infection model of necrotic enteritis (NE) using *EM* and NetB^+^ *Clostridium perfringens* (*CP*). A total of 432 fourteen-d-old male Cobb 500 broiler chickens were divided into 6 groups with 6 replicates each. The six different groups were as follows: Control, non-challenged; T0^+^, challenged with *CP* at 1 × 10^9^ colony forming unit; T5K^+^, T0^+^ + 5,000 *EM* oocysts; T10K^+^, T0^+^ + 10,000 *EM* oocysts; T20K^+^; T0^+^ + 20,000 *EM* oocysts; and T40K^+^; T0^+^ + 40,000 *EM* oocysts. The challenge groups were orally inoculated with *EM* strain 41A on d 14, followed by NetB^+^ *CP* strain Del-1 on 4 days post inoculation (dpi). Increasing *EM* oocysts decreased d 21 body weight, body weight gain, feed intake (linear and quadratic, *p* < 0.001), and feed efficiency (linear, *p* < 0.001) from 0 to 7 dpi. Increasing *EM* oocysts increased jejunal NE lesion score and intestinal permeability on 5, 6, and 7 dpi (linear, *p* < 0.05). On 7 dpi, increasing the infection doses of *EM* oocysts increased jejunal *CP* colony counts (linear, *p* < 0.05) and increased fecal *EM* oocyst output (linear and quadratic, *p* < 0.001). Furthermore, increasing the infection doses of *EM* oocysts decreased the villus height to crypt depth ratios and the goblet cell counts (linear, *p* < 0.05) on 6 dpi. Increasing *EM* oocysts downregulated the expression of MUC2, B^0^AT, B^0,+^AT, PepT1, GLUT2, AvBD3 and 9, LEAP2, and TLR4, while upregulating CLDN1, CATHL3, IL-1β, IFN-γ, TNFSF15, TNF-α, IL-10, and Gam56 and 82 on 6 dpi (linear, *p* < 0.05). Additionally, increasing *EM* oocysts decreased Pielou’s evenness and Shannon’s entropy (linear, *p* < 0.01). In conclusion, increasing the infection doses of *EM* significantly aggravated the severity of NE and exerted negative impact on intestinal health from 5 to 7 dpi.

## 1 Introduction

Necrotic enteritis (NE) is an enteric disease of poultry, primarily caused by the Gram-positive NE-B like toxin (NetB) positive bacterium, *Clostridium perfringens* (Lee and Lillehoj, 2021). According to a 2015 report, the global annual loss from NE amounts to approximately 6 billion USD (Wade and Keyburn, 2015). *C. perfringens* is a normal flora in the chicken gut (Paiva and McElroy, 2014), but several predisposing factors have been shown to induce NE (Moore, 2016). These factors include coccidiosis (Paiva and McElroy, 2014), viral infections (Lee et al., 2011), heat and cold stress (Calefi et al., 2014; Tsiouris et al., 2015b), high stocking density (Tsiouris et al., 2015a), changes in gastrointestinal pH (Williams, 2005), high non-starch polysaccharide diets (Annett et al., 2002), wet litter (Hermans and Morgan, 2007), and high crude protein diets such as fishmeal (Cooper and Songer, 2010). Among these factors, coccidiosis is recognized as a crucial predisposing factor in the pathogenesis of NE (Lee et al., 2011) because *Eimeria* parasites undergo an intracellular development in the gut which directly impair the intestinal mucosa (Lee et al., 2023). Specifically, *E. maxima* is considered as an important factor in the occurrence of NE (Adhikari et al., 2020) causing physical damage to the intestinal epithelium of chickens, creating favorable conditions for the colonization and proliferation of *C. perfringens* (Van Immerseel et al., 2009), and the leaked plasma proteins serve as nutrients for *C. perfringens* (Van Immerseel et al., 2004). The physical damage inflicted by coccidiosis exposes certain types of collagen in the extracellular matrix (Wade et al., 2015; Lepp et al., 2021), providing binding sites for *C. perfringens* which led to the production of major toxins such as NetB (Arora et al., 2021; Goo et al., 2023b). Furthermore, the damaged epithelium triggers increased mucus production as a protective response, which creates a protein-rich environment for the growth and proliferation of *C. perfringens* on the damaged intestinal surface (Collier et al., 2008).

There are two types of NE: clinical and subclinical. Clinical NE is characterized by high mortality, diarrhea, and depression (Songer and Meer, 1996; McDevitt et al., 2006). On the other hand, subclinical NE exhibits reduced growth performance without mortality. It can be diagnosed by reduced feed efficiency and intestinal lesions. In the field, most of the economic losses attributed to NE are due to subclinical infections (Shojadoost et al., 2012). Due to the current trend of limiting antibiotic use, there is a growing interest in understanding the pathology of NE and exploring antibiotic-alternative approaches for its prevention (Prescott et al., 2016). Therefore, it is crucial to develop an experimental NE model capable of reproducing the subclinical symptoms caused by NE. *E. maxima-*induced coccidiosis mainly damages the jejunum, and has been widely employed in many NE studies (Williams et al., 2003; Park et al., 2008; Hong et al., 2012; Bortoluzzi et al., 2019; Lee et al., 2021). Many other predisposing factors for generating experimental NE, such as temperature, diets, viruses, and others, often yield inconsistent results, making it difficult to interpret data from such models.

When attempting to create an experimental NE using *E. maxima* as a predisposing factor, several key aspects should be considered. These include: 1) the source or origin of the *E. maxima* strains, 2) the potential interference or interaction with other *Eimeria* species that mainly target the small intestine, such as *E. acervulina* or *E. necatrix*, 3) the pathogenicity of *E. maxima*, including factors like storage duration after sporulation or storage conditions, 4) the timing of challenge before the *C. perfringens* challenge, and 5) the dose of *E. maxima* oocysts. Establishing these key aspects is crucial to establish reproducible and reliable experimental NE models using *E. maxima* as a predisposing factor for *C. perfringens* infection. Among these key factors, the dose of *E. maxima* has been identified as the most important factor in establishing reliable experimental NE models (Shojadoost et al., 2012). Caution should be exercised when selecting the dose of *Eimeria* because it can cause serious intestinal lesions that can lead to death (Prescott et al., 2016). Additionally, using high doses can result in severe experimental NE, which may distort or mask the beneficial effects of interventions such as feed additives or immunization (Shojadoost et al., 2012). Therefore, it is essential to use an optimum *E. maxima* dose to experimentally evaluate various intervention strategies aimed at alleviating the detrimental effects of NE in poultry. While it is generally accepted to use 0.5–5 × 10^4^ *E. maxima* oocysts for successful experimental NE models (Williams et al., 2003; Wang et al., 2019; Whelan et al., 2019; Park et al., 2022), specific data on various parameters for different doses of *E. maxima* are still insufficient. Some coccidiosis experiments have reported that key indicators do not linearly decrease or increase with changes in *E. maxima* oocyst dose (Williams, 2001; Sakkas et al., 2018), presumably due to the crowding effect (Williams, 2001). If the threshold is exceeded, the reproductive efficiency decreases (Williams, 2001).

Our hypothesis was that as the challenge level of *E. maxima* oocysts increases, the impact of NE caused by *C. perfringens* also increases, leading to decreased productivity, deterioration of intestinal health, and changes in intestinal immunity and microbiota compositions. Additionally, it is expected that as the dose of infecting *E. maxima* increases, certain parameters related to NE infection may not respond linearly, as there will be an optimal *Eimeria* dosage level that produces the most detrimental effects of NE. Therefore, the objectives of this study were: 1) to generate an experimental subclinical NE using our co-infection *E. maxima/C. perfringens* model suitable for feed additive studies, and 2) to investigate the effects of subclinical NE on growth performance, intestinal health and immune parameters, and changes in intestinal microbiota in broilers.

## 2 Materials and methods

### 2.1 Chickens, experimental design, and sample collections

The experiment was conducted at the Poultry Research Center, University of Georgia and approved by the Institutional Animal Care and Use Committee (A2020 01-018). Animal husbandry followed the Cobb 2018 nutritional and management guidelines. A total of 720 0-d-old Cobb 500 male broiler chickens were raised and fed until 14 d of age. On d 14, a total of 432 chickens with an average body weight (BW: 424.0 ± 2.9 g) were assigned to 6 treatment groups with 6 replicates of 12 chickens per battery cage. The six treatments in this experiment were as follows: 1) Control: non-challenged group; 2) T0^+^: challenged with 1 × 10^9^ *C. perfringens* colony forming units (cfu) on d 18; 3) T5K^+^: challenged with 5,000 *E. maxima* oocysts on d 14 and 1 × 10^9^ *C. perfringens* cfu on d 18; 4) T10K^+^: challenged with 10,000 *E. maxima* oocysts on d 14 and 1 × 10^9^ *C. perfringens* cfu on d 18; 5) T20K^+^: challenged with 20,000 *E. maxima* oocysts on d 14 and 1 × 10^9^ *C. perfringens* cfu on d 18; and 6) T40K^+^: challenged with 40,000 *E. maxima* oocysts on d 14 and 1 × 10^9^ *C. perfringens* cfu on d 18. All the chickens were raised in nipple-installed 4-layer battery cages (40 × 14 × 16 inches). The diet formulations are provided in Table 1. Two phase diets (Phase 1: 21% crude protein diet from d 0 to 18; Phase 2: 24% crude protein diet from d 19 to 28) were formulated using corn-soybean meal-based mash form. Feed and water were provided *ad libitum*. Daily calculations were made for feed intake (FI) and mortality. Body weights (BW) of chickens per cage were measured on d 14, 18, 21, and 28 to calculate the body weight gain (BWG) and feed conversion ratio (FCR). On 5, 6, 7, and 14 days post inoculation of *E. maxima* (dpi), two chickens per cage were euthanized by cervical dislocation to measure intestinal permeability and NE lesion scores. On 6 dpi, the jejunum and ileum were collected and directly fixed in a 10% formalin solution for intestinal morphology measurement. Jejunal tissues and cecal contents were collected and immediately stored at −80°C for gene expression and microbiome analysis. On 7 dpi, jejunal digesta were collected to measure *C. perfringens* colony counts and apparent ileal digestibility. Fresh fecal samples were also collected from the cages on 5, 6, 7, and 14 dpi and stored at 4°C for *E. maxima* oocyst enumeration.

**TABLE 1:** Composition of Cobb 500 broiler diets (as-fed basis, %).

Ingredients, %	Phase^a^ 1 d 14–18	Phase 2 d 19–28
Corn, grain	58.58	53.61
Soybean meal—46%	31.72	39.61
Soybean oil	2.76	3.69
Sand	2.00	0.00
Dicalcium phosphate	1.69	1.24
Salt	1.38	0.35
Limestone	1.16	0.99
L-Lys HCl	0.28	0.00
DL-Met	0.15	0.08
Thr	0.15	0.00
Mineral premix^b^	0.08	0.08
Vitamin premix^c^	0.05	0.05
Titanium dioxide	0.00	0.30
Total	100.0	100.0
Calculated value		
ME (kcal/kg)	3,000	3,100
Crude protein, %	21.00	24.00
Total Ca, %	0.90	0.76
Available P, %	0.45	0.38
Lys	1.20	1.20
Met	0.45	0.42
TSAA	0.85	0.73
Analyzed value		
Crude protein, %	20.93	24.15

^a^ Phase 1, corn-soybean meal-based 21% crude protein diet; Phase 2, corn-soybean meal-based 24% crude protein diet.

^b^ Mineral premix per kg of diet: Mn, 100.5 mg; Zn, 80.3 mg; Ca, 24 mg; Mg, 20.1 mg; Fe, 19.7 mg; Cu, 3 mg; I, 0.75 mg; Se, 0.30 mg.

^c^ Vitamin premix per kg of diet: vitamin A, 3,527 IU; vitamin D_3_, 1,400 IU; vitamin E, 19.4 IU; niacin, 20.28 mg; D-pantothenic acid, 5.47 mg; riboflavin, 3.53 mg; vitamin B_6_, 1.46 mg; menadione, 1.10 mg; thiamin, 0.97 mg; folic acid, 0.57 mg; biotin, 0.08 mg; vitamin B_12_, 0.01 mg.

### 2.2 Necrotic enteritis model and lesion score

The NE model in this experiment was established based on the previously reported experiments (Park et al., 2022; Goo et al., 2023a) with slight modifications. On d 14, all chickens were orally inoculated with 1 mL of the corresponding doses of *E. maxima* (Strain 41A from USDA-ARS) oocysts (0, 5,000, 10,000, 20,000, and 40,000) and followed by the administration of 1 mL of phosphate-buffered saline (PBS) for the Control group or NetB-positive *C. perfringens* Del-1 at 1 × 10^9^ cfu for the challenged groups on d 18. On d 18, the feeds were switched to a 24% crude protein Phase 2 diet prior to the challenge with *C. perfringens*, in order to facilitate NE pathogenesis (Park et al., 2022). On 5, 6, 7, and 14 dpi, two chickens per cage were euthanized to measure the jejunal NE lesion scores. The NE lesions were scored on a 4-scale system ranging from 0 (no lesion) to 3 (severe lesion), with slight modifications (Park et al., 2008; Lee et al., 2021). The NE lesion measurement was conducted in approximately 20 cm of the jejunum from Meckel’s diverticulum. The NE lesion scores were assessed through a blinded method by two independent investigators.

### 2.3 Intestinal permeability

Intestinal permeability was measured on 5, 6, 7, and 14 dpi using fluorescein isothiocyanate-dextran (FITC-d, Molecular weight 4,000; Sigma-Aldrich, Canada), following previous experiments (Teng et al., 2020) with slight modifications. In brief, a FITC-d solution with a concentration of 2.2 mg/mL was prepared using PBS under dark condition. The solution was then orally administered to one chicken per cage. Two hours after administration, blood samples were collected and stored in a completely dark room for 2 h. The samples were then centrifuged at 2,000 × g for 12 min to collect sera. To determine the FITC-d level, a standard curve was generated by a serial dilution of sera extracted from 5 non-experimental chickens. Hundred microliters of serum samples were transferred to 96 flat-bottom dark-well plates, and fluorescence was measured at an optical density (OD) of 485/525 nm using a Spectra Max 5 microplate reader (Molecular Devices, Sunnyvale, CA).

### 2.4 Jejunal *C. perfringens* colony counting

On 7 dpi (d 21), one chicken per cage was euthanized, and approximately 20 g of jejunal contents (lower jejunal to upper ileal) were collected in sterile Whirl-Pak filter bags (Nasco, Fort Atkinson, WI). To each filter bag containing the jejunal content, 10 mL of buffered peptone water (BPW; Himedia, Mumbai, India) was added, and the samples were homogenized using a Masticator Silver Panoramic (Neutec Group Inc., Farmingdale, NY) for 1 min. Following homogenization, 1 mL of the mixed jejunal contents was transferred to a sterile glass dilution tube and diluted to 10^−8^ by serial dilution with BPW. Subsequently, 100 µL of the diluted contents (10^−2^, 10^−4^, 10^−6^, and 10^−8^) were plated on Tryptose Sulfite Cycloserine and Shahadi Ferguson Perfringens (TSC/SFP; Oxoid Ltd., Hampshire, United Kingdom) agar. The plates were incubated at 37°C for 24 h under anaerobic conditions created using an anaerobic gas-pack system (AnaeroPack™, Thermo Scientific, MA).

### 2.5 Fecal *E. maxima* oocyst counting and water content

On 4, 5, 6, and 13 dpi (1 d before feces collection), clean trays were placed under the cages to collect *E. maxima* oocysts. Each day, approximately 150 g of fecal samples were collected from homogenized fresh feces and stored at 4°C for further analysis. The fecal samples were divided into two sets of 75 g each. One set was used to measure the moisture content at 70°C in a drying oven, while the other set was used for *E. maxima* oocyst counting. The oocyst counts were conducted with slight modification to a previously described method (Choi et al., 2022b). Briefly, 5 g of fecal samples was mixed with 30 mL of tap water and vigorously vortexed. Then, 1 mL of the feces sample was mixed with 10 mL (1:11) of saturated salt solution and vortexed again. Subsequently, 650 µL of the feces mixture was added to a McMaster chamber (Vetlab Supply, Palmetto Bay, FL), and the *E. maxima* oocysts were counted. The total *E. maxima* oocysts per gram of feces were expressed as log_10_.

### 2.6 Ileal digestibility and water content

The Phase 2 feed samples (24% crude protein) and ileal digesta were dried in an oven at 70°C and subsequently ashed overnight at 600°C. The concentration of titanium dioxide (TiO_2_) in each sample was determined following the method described by Short et al. (1996). The analysis of crude protein concentration was performed using nitrogen combustion analysis according to the AOAC (2000) analysis method 942.05. The apparent ileal digestibility (AID) of dry matter (DM), organic matter (OM), crude protein, and ash was calculated based on these measurements.

### 2.7 Intestinal morphology and goblet cell counting

On 6 dpi (d 20), jejunum and ileum tissues were collected from euthanized chickens and immediately fixed in a 10% formalin solution. To measure the villus height (VH) to crypt depth (CD) ratio (VH:CD) and goblet cell (GC) numbers per villus, the fixed intestinal sections were subjected to Hematoxylin counterstained Period acid-Schiff (PAS) staining, following the method described by Liu et al. (2022). The stained sections of the intestine were then visualized using a BZ microscope (BZ-X810, Keyence, Osaka, Japan). VH and CD were measured at a magnification of ×4, while GC counting as conducted at a magnification of ×10. The captured images were analyzed using the ImageJ program (National Institute of Health, Bethesda, MD).

### 2.8 qRT-PCR analysis

For RNA extraction and cDNA synthesis, 100 mg of jejunal samples were homogenized with 1 mL of QIAzol lysis reagent (Qiagen, Valencia, CA) using a bead beater (Biospec Products, Bartlesville, OK). The RNA was then extracted following the manufacturer’s protocol, and its quantity and purity were assessed using a NanoDrop 2000 spectrophotometer (Thermo Scientific, Waltham, MA). Subsequently, cDNA synthesis was performed using high-capacity cDNA synthesis kits (Applied Biosystems, Foster City, CA) according to the manufacturer’s instructions. Quantitative real-time reverse transcriptase polymerase chain reaction (qRT-PCR) was conducted using SYBR Green Master Mix on a Step One thermocycler (Applied Biosystems, Foster City, CA). The final reaction volume for qRT-PCR was 10 μL, consisting of 5 µL of SYBR Green Master Mix, 1.5 µL of cDNA, 0.5 µL of forward primer, 0.5 µL of reverse primer, and 2.5 µL of nuclease-free water. The thermal cycles for all reactions were as follows: polymerase activation and DNA denaturation for 5 min at 95°C, followed by 40 cycles of denaturation at 95°C for 15 s, and annealing/extension at 60°C for 1 min. After amplification, a melting curve analysis was performed by collecting fluorescence data. Beta-actin and 18 s *E. maxima* ribosomal RNA were used as reference genes for jejunal samples and *E. maxima*, respectively. The relative fold changes of gene expression levels were determined using the 2^−ΔΔCt^ method compared to the Control. The primer sequences used in this study are provided in Table 2.

**TABLE 2:** Primers used for qRT-PCR.

Primers^a^	Forward sequence	Reverse sequence	Product size, bp	Accession number
Tight junction proteins				
CLDN1	TGG​AGG​ATG​ACC​AGG​TGA​AGA	CGA​GCC​ACT​CTG​TTG​CCA​TA	115	NM_001013611.2
JAM2	AAG​GAT​TCT​GGG​ACC​TAC​CG	GTT​CCC​GTC​ATT​GCA​GAG​TT	143	NM_001397141.1
OCLN	GTT​GGA​TGA​GTC​CCA​GTA​TG	GTC​GAA​CTC​CTG​CTT​GTA​G	129	NM_205128.1
ZO2	GAA​AGC​AGA​CCC​TGC​TCA​AC	TGG​ATG​AAT​GCA​AAT​CCA​GA	141	NM_001396726.1
Mucin				
MUC2	ATG​CGA​TGT​TAA​CAC​AGG​ACT​C	GTG​GAG​CAC​AGC​AGA​CTT​TG	110	JX284122.1
Nutrient transporters				
B^0^AT	TCT​ATT​GAA​GAT​TCG​GGC​AC	AAT​GGT​AAG​CAC​AAG​GTA​TGG	153	XM_419056.8
B^0,+^AT	ACT​GGG​ATT​GTT​CTC​CTT​GC	CCA​TCA​TAT​GCC​CAG​AGT​CC	122	NM_001199133.2
EAAT3	TGC​TGC​TTT​GGA​TTC​CAG​TGT	AGC​AAT​GAC​TGT​AGT​GCA​GAA​GTA​ATA​TAT​G	79	XM_424930.8
PepT1	ATA​CAA​TTG​GGC​AGG​CAG​TC	GCG​ATG​AGA​ATC​AAG​CCA​GT	127	NM_204365.2
GLUT2	TCA​TTG​TAG​CTG​AGC​TGT​T	CGA​AGA​CAA​CGA​ACA​CAT​AC	147	NM_207178.2
Host defense peptides				
AvBD3	CCA​CCC​AGT​GCA​GAA​TAA​GA	GCT​CTT​CCA​CAG​CAG​GAA​AT	106	NM_204650.2
AvBD9	GCT​GAC​ACC​TTA​GCA​TGC​AG	CAT​TTG​CAG​CAT​TTC​AGC​TT	113	NM_001001611.3
CATHL3	GCT​GTG​GAC​TCC​TAC​AAC​CA	CCA​TGA​TGG​TGA​AGT​TGA​GG	124	NM_001311177.2
LEAP2	TAT​TCT​TCT​CGC​TGC​TGC​TC	AGG​CTC​CAA​CAG​GTC​TCA​GT	123	NM_001001606.2
TLR/NF-κB signaling pathway-related genes				
NF-κB	GAA​GGA​ATC​GTA​CCG​GGA​ACA	CTC​AGA​GGG​CCT​TGT​GAC​AGT​AA	131	NM_205134.1
TLR2	CGGTGGAAAGGGAGAAAG	CTT​GCC​ACA​TCA​GCT​TCA​TT	103	NM_001397379.1
TLR4	CCT​GGA​CTT​GGA​CCT​CAG​TT	TTG​TAT​GGA​TGT​GGC​ACC​TT	110	NM_001030693.2
Cytokines				
IL-1β	CCT​TCA​CCC​TCA​GCT​TTC​AC	CCC​TCC​CAT​CCT​TAC​CTT​CT	138	NM_204524.2
IL-2	TGC​AGT​GTT​ACC​TGG​GAG​AA	CTT​GCA​TTC​ACT​TCC​GGT​GT	149	NM_204153.2
IFN-γ	GGC​GTG​AAG​AAG​GTG​AAA​GA	TCC​TTT​TGA​AAC​TCG​GAG​GA	133	NM_205149.2
TNFSF15	TTC​TGA​AGC​AGC​GAG​CAG​TA	TTG​TCT​TCC​CAC​TGC​AGG​AT	139	NM_001024578.2
TNF-α	TTC​AGA​TGA​GTT​GCC​CTT​CC	TCA​GAG​CAT​CAA​CGC​AAA​AG	150	NM_204267.2
IL-6	GCT​ACA​GCA​CAA​AGC​ACC​TG	GAC​TTC​AGA​TTG​GCG​AGG​AG	112	NM_204628.2
IL-10	GCT​GCG​CTT​CTA​CAC​AGA​TG	CTC​CTC​TTC​TCG​CAG​GTG​AA	150	NM_001004414.4
*E. maxima* gametocytes				
APN	TTT​CGC​CGT​TGA​TTC​TGT​AG	CTC​CCC​ATT​CAA​GAC​CAA​GT	124	XM_013483142.1
EF2	GAT​GGA​AAG​GGA​GAA​CAG​GA	ACA​GAA​TCC​ACG​ACG​ACA​AG	125	XM_013477319.1
Gam56	CTT​CCC​TGA​AAC​CCC​TAT​GA	TGA​GGC​TAC​GAA​ATG​TGA​GC	121	AY129951.2
Gam82	AGG​TAC​CCC​AGC​TAT​GAT​GC	CAC​GCG​AGT​ATA​TGC​TGG​AT	109	AY179510.2
IMC1	CAT​ACC​AGC​CAG​TTG​ACA​CC	CTT​GGG​AAC​CTC​CAC​AAT​CT	113	XM_013481172.1
References				
ACTB	CAA​CAC​AGT​GCT​GTC​TGG​TGG​TA	ATC​GTA​CTC​CTG​CTT​GCT​GAT​CC	205	NM_205518.2
Em18S	TCG​CGT​CTC​TAA​TGA​TCG​TC	TCT​GCA​ATT​CAC​AAT​GCG​TA	110	JN113574.1

^a^ Primers: CLDN1, claudin 1; JAM2, junctional adhesion molecule 2; OCLN, occludin; ZO2, zonula occludens 2; MUC2, mucin 2; B0AT, sodium dependent amino acid transporter (solute carrier family 6 member 19, SLC6A19); B0,+AT, sodium independent amino acid transporter (solute carrier family 7 member 9, SLC7A9); EAAT3, excitatory amino acid transporter 3 (solute carrier family 1 member 1, SLC1A1); PepT1, peptide transporter 1 (solute carrier family 15 member 1, SLC15A1); GLUT2, glucose transporter 2 (solute carrier family 2 member 2, SLC2A2); AvBD, avian beta-defensin; CATHL3, cathelicidin 3; LEAP2, liver-expressed antimicrobial peptides 2; NF-κB, nuclear factor kappa B p 65; TLR, toll-like receptor; IL, interleukin; IFN-γ, interferon gamma; TNFSF15, tumor necrosis factor superfamily 15; TNF-α, tumor necrosis factor alpha; APN, *E. maxima* aminopeptidase N; EF2, elongation factor 2; Gam, *E. maxima* gametocyte antigen; IMC1, membrane skeletal protein inner membrane complex 1; ACTB, beta-actin; Em18S, *E. maxima* ribosomal RNA.

### 2.9 Microbiome analysis

For the cecal microbiome analysis, DNA was extracted from approximately 100 mg of cecal contents using QIAamp^®^ DNA stool mini kits (Qiagen GmbH, Hilden, Germany) following the manufacturer’s protocol. After DNA extraction, the quality and quantity of the DNA were assessed using a NanoDrop 2000 spectrophotometer (Thermo Scientific, Waltham, MA). The samples were then sent to LC Science (Houston, TX) for 16s rRNA gene sequencing, following previous experimental methods (Choi et al., 2022a). The 16s rRNA gene sequences were processed and analyzed using Quantitative Insights into Microbial Ecology 2 (QIIME2) version 2022.02, as described by Bolyen et al. (2019). Alpha-diversity measurements, including Faith’s phylogenetic diversity, observed richness, Pielou’s evenness, and Shannon’s entropy, were analyzed using QIIME2’s built-in functions. Beta-diversity was assessed through Principal Coordinate Analysis (PCoA) plots, which visualized similarities between the Control and challenged groups using unweighted UniFrac distance, weighted UniFrac distance, Jaccard distance, and Bray-Curtis dissimilarity. Taxonomy bar-plots at the phylum and family levels were generated using RStudio software (R Version 4.2.2, RStudio PBC, Boston, MA).

### 2.10 Statistical analysis

All statistical analyses were conducted using RStudio software (R Version 4.2.2, RStudio PBC, Boston, MA). Tukey’s honestly significant differences (HSD) test was used to determine the differences between all treatment groups when the *p*-value was less than 0.05 (*p* < 0.05). Orthogonal polynomial contrast tests were performed to assess the linear and quadratic effects of the five different *E. maxima* level groups (T0^+^, T5K^+^, T10K^+^, T20K^+^, and T40K^+^) excluding the NC group. Because the intervals between the treatment groups were not constant (5,000, 5,000, 10,000, and 20,000), the orthogonal polynomial coefficients were calculated as follows: linear (−0.4743, −0.3162, −0.1581, 0.1581, and 0.7905) and quadratic (0.5460, 0.0352, −0.3346, −0.6516, and 0.4050). For the *E. maxima* gametocytes gene expression analysis, which included only the *E. maxima* challenged groups (T5K^+^, T10K^+^, T20K^+^, and T40K^+^), the orthogonal polynomial coefficients were recalculated as follows: linear (−0.5129, −0.3264, 0.0467, and 0.7926) and quadratic (0.5296, −0.1059, −0.7680, and 0.3443). Beta-diversity matrices were analyzed using permutation multivariate analysis of variance (PERMANOVA) with 999 permutations, and PCoA was performed on these dissimilarity indices. Segmented linear regression analysis was used to determine the maximal effects of *E. maxima* in the current NE coinfection experiment for parameters that exhibited a quadratic relationship Table 3.

**TABLE 3:** A summary of the results of items that exhibit maximum or optimal results from d 14 to 21 (0–7 dpi).

	Maximal effect^a^ of *E. maxima* dose	Optimal effect^b^ (X0) of *E. maxima* dose	Linear and quadratic effects^c^
BW (4 dpi^d^)	40,000	NA	Linear (↓)
BW (7 dpi)	40,000	16,085 (R^2^ = 0.83)	Linear (↓) and quadratic
BWG (0–7 dpi)	40,000	16,530 (R^2^ = 0.83)	Linear (↓) and quadratic
FI (0–7 dpi)	40,000	14,662 (R^2^ = 0.91)	Linear (↓) and quadratic
FCR (0–7 dpi)	40,000	NA	Linear (↑)
Jejunal NE lesion scores (5 dpi)	20,000 and 40,000	14,782 (R^2^ = 0.94)	Linear (↑) and quadratic
Jejunal NE lesion scores (6 dpi)	20,000	NA	Linear (↑)
Jejunal NE lesion scores (7 dpi)	40,000	NA	Linear (↑)
Intestinal permeability (5 dpi)	40,000	NA	Linear (↑)
Intestinal permeability (6 dpi)	40,000	10,598 (R^2^ = 0.54)	Linear (↑) and quadratic
Jejunal *C. perfringens* colony count (7 dpi)	40,000	NA	Linear (↑)
Fecal *E. maxima* oocyst count (6 dpi)	40,000	5,389 (R^2^ = 0.98)	Linear (↑) and quadratic
Fecal *E. maxima* oocyst count (7 dpi)	40,000	5,645 (R^2^ = 0.97)	Linear (↑) and quadratic
Apparent ileal digestibility of DM	10,000	10,550 (R^2^ = 0.48)	Linear (↓) and quadratic
Apparent ileal digestibility of ash	10,000	10,000 (R^2^ = 0.44)	Linear (↓) and quadratic
Apparent ileal digestibility of CP	10,000	10,000 (R^2^ = 0.40)	Linear (↓) and quadratic
Ileal digesta water content	40,000	NA	Linear (↑)
Jejunal VH:CD	40,000	4,555 (R^2^ = 0.65)	Linear (↓) and quadratic
Jejunal goblet cell count	20,000 and 40,000	NA	Linear (↓)
Ileal VH:CD	40,000	NA	Linear (↓)
Ileal goblet cell count	40,000	19,389 (R^2^ = 0.52)	Linear (↓) and quadratic
The gene expression of *CLDN1*	40,000	NA	Linear (↑)
The gene expression of *OCLN*	NA	13,180 (R^2^ = 0.28)	Quadratic
The gene expression of *ZO2*	NA	13,691 (R^2^ = 0.49)	Quadratic
The gene expression of *MUC2*	20,000	6,690 (R^2^ = 0.70)	Linear (↓) and quadratic
The gene expression of *B* ^*0,+*^ *AT*	20,000	11,952 (R^2^ = 0.45)	Linear (↓) and quadratic
The gene expression of *EAAT3*	NA	12,501 (R^2^ = 0.32)	Quadratic
The gene expression of *PepT1*	NA	7,981 (R^2^ = 0.55)	Quadratic
The gene expression of *GLUT2*	20,000	5,631 (R^2^ = 0.59)	Linear (↓) and quadratic
The gene expression of *AvBD9*	40,000	NA	Linear (↓)
The gene expression of *LEAP2*	20,000	6,343 (R^2^ = 0.75)	Linear (↓) and quadratic
The gene expression of *TLR4*	NA	14,587 (R^2^ = 0.45)	Linear (↓) and quadratic
The gene expression of *IL-1β*	20,000	NA	Linear (↑)
The gene expression of *IFN-γ*	40,000	NA	Linear (↑)
The gene expression of *IL-10*	40,000	NA	Linear (↑)
The gene expression of *Gam82*	20,000	22,465 (R^2^ = 0.39)	Linear (↑) and quadratic
Pielou’s evenness	40,000	NA	Linear (↓)
Shannon’s entropy	40,000	NA	Linear (↓)
The relative frequency of *Actinobacteria*	NA	13,181 (R^2^ = 0.31)	Linear (↓) and quadratic
The relative frequency of *Lachnospiraceae*	NA	20,000 (R^2^ = 0.22)	Quadratic

^a^ The maximal effect of *E. maxima* oocyst dose was determined using Tukey’s honestly significant differences test with a significance level of *p* < 0.05.

^b^ In cases where a quadratic relationship was observed, a segmented linear regression analysis was conducted to determine the optimal effect of *E. maxima* oocyst dose (X0), which represents the point of intersection between the two segments.

^c^ Linear and quadratic effects were assessed using an orthogonal polynomial contrast test, except for the Control group.

Abbreviations: NA, not applicable; BW, body weight; BWG, body weight gain; FI, feed intake; FCR, feed conversion ratio; NE, necrotic enteritis; DM, dry matter; CP, crude protein; VH:CD, villus height to crypt depth ratio; CLDN1, claudin 1; OCLN, occludin; ZO2, zonula occludens 2; MUC2, mucin 2; B^0,+^AT, sodium independent amino acid transporter (solute carrier family 7 member 9, SLC7A9); EAAT3, excitatory amino acid transporter 3 (solute carrier family 1 member 1, SLC1A1); PepT1, peptide transporter 1 (solute carrier family 15 member 1, SLC15A1); GLUT2, glucose transporter 2 (solute carrier family 2 member 2, SLC2A2); AvBD9, avian beta-defensin 9; LEAP2, liver-expressed antimicrobial peptides 2; TLR4, toll-like receptor 4; IL-1β, interleukin 1 beta; IFN-γ, interferon gamma; IL-10, interleukin 10; Gam82, *E. maxima* gametocyte antigen 82 kDa.

^d^ dpi, days post inoculation of *E. maxima*.

## 3 Results

### 3.1 Growth performance, mortality, and daily feed intake

Growth performance data are presented in Table 4. No mortality occurred in any groups during the entire experimental period from 0 to 14 dpi. On 4 dpi, the T40K^+^ group showed decreased BW compared to the Control group (*p* < 0.05). At 7 dpi, both the T20K^+^ and T40K^+^ groups showed significantly lower BW compared to the Control, T0^+^, T5K^+^, and T10K^+^ groups (*p* < 0.001), while the T10K^+^ group showed decreased BW compared to the Control and T0^+^ groups (*p* < 0.001). At 14 dpi, the T20K^+^ and T40K^+^ groups showed significantly lower BW compared to the Control and T0^+^ groups (*p* < 0.001), and the T10K^+^ group showed lower BW compared to the Control group (*p* < 0.001). However, there were no significant differences in BW between the T20K^+^ and T40K^+^ groups throughout the entire experimental period. Overall, the T40K^+^ group exhibited the lowest BW among all groups. Among the coinfection groups (T0^+^, T5K^+^, T10K^+^, T20K^+^, and T40K^+^), increasing *E. maxima* levels with *C. perfringens* coinfection resulted in a significant decrease in BW on 4 dpi (linear, *p* < 0.01), 7 dpi (linear and quadratic, *p* < 0.001), and 14 dpi (linear, *p* < 0.001). Regarding the BWG from 0 to 7 dpi, the T10K^+^, T20K^+^ and T40K^+^ groups showed decreased BWG compared to the Control and T0^+^ groups (*p* < 0.001), whereas the T5K^+^ and T10K^+^ groups had significantly higher BWG than the T20K^+^ and T40K^+^ groups (*p* < 0.001). The T5K^+^, T10K^+^, T20K^+^, and T40K^+^ groups showed decreased FI (*p* < 0.001), and the T20K^+^ and T40K^+^ groups had a higher FCR (*p* < 0.001) compared to the Control and T0^+^ groups from 0 to 7 dpi. However, there were no significant differences in BWG, FI, and FCR among the treatments from 8 to 14 dpi. Over the entire period (0–14dpi), the T20K^+^ and T40K^+^ groups showed decreased BWG (*p* < 0.001) and FI (*p* < 0.01), and the T40K^+^ group showed the highest FCR (*p* < 0.01) compared to the Control and T0^+^ groups. From 0 to 14 dpi, increasing *E. maxima* levels with *C. perfringens* coinfection linearly decreased BWG (linear, *p* < 0.001) and FI (linear, *p* < 0.01) and increased FCR (linear, *p* < 0.01). Overall, the T40K^+^ group showed the worst BWG, FI, and FCR among all groups.

**TABLE 4:** Effects of different infecting doses of *E. maxima* oocyst on growth performance in NE-infected broiler chickens from d 14 to 28 (0 to 14 dpi).

	Treatments^1^						SEM (*n* = 6)	*P*-value^2^		
	Control	T0^+^	T5K^+^	T10K^+^	T20K^+^	T40K^+^		ANOVA	L	Q
BW, g										
d 14 (0 dpi^3^)	423	425	423	423	425	425	1.2	0.888	0.480	0.620
d 18 (4 dpi)	643^a^	637^ab^	636^ab^	644^a^	625^ab^	620^b^	5.0	<0.05	<0.01	0.746
d 21 (7 dpi)	864^a^	869^a^	821^ab^	769^b^	702^c^	670^c^	14.6	<0.001	<0.001	<0.001
d 28 (14 dpi)	1,474^a^	1,409^ab^	1,277^bc^	1,257^bc^	1,194^c^	1,115^c^	41.4	<0.001	<0.001	0.119
BWG, g										
0–7 dpi	440^a^	444^a^	398^ab^	346^b^	277^c^	245^c^	13.9	<0.001	<0.001	<0.001
8–14 dpi	611	540	456	488	491	446	36.6	0.143	0.215	0.832
0–14 dpi	1,051^a^	984^ab^	854^bc^	834^bc^	769^c^	691^c^	40.9	<0.001	<0.001	0.119
FI, g										
0–7 dpi	634^a^	644^a^	588^b^	558^b^	506^c^	480^c^	7.8	<0.001	<0.001	<0.001
8–14 dpi	956	935	867	860	853	850	40.3	0.521	0.286	0.338
0–14 dpi	1,590^a^	1,580^a^	1,455^ab^	1,418^ab^	1,359^b^	1,330^b^	43.0	<0.01	<0.01	0.055
FCR, g/g										
0–7 dpi	1.44^c^	1.45^c^	1.49^c^	1.63^bc^	1.83^ab^	2.01^a^	0.066	<0.001	<0.001	0.256
8–14 dpi	1.58	1.78	1.91	1.78	1.77	1.98	0.119	0.396	0.360	0.444
0–14 dpi	1.52^b^	1.61^b^	1.71^ab^	1.72^ab^	1.79^ab^	1.97^a^	0.069	<0.01	<0.01	0.987

^a–c^Means in the same row with different superscripts indicate statistical differences (*P* < 0.05).

1 Control, non-challenged control; T0^+^, challenged with 1 × 10^9^ *C. perfringens* cfu on d 18; T5K^+^, challenged with 5,000 *E. maxima* oocysts on d 14 and 1 × 10^9^ *C. perfringens* cfu on d 18; T10K^+^, challenged with 10,000 *E. maxima* oocysts on d 14 and 1 × 10^9^ *C. perfringens* cfu on d 18; T20K^+^, challenged with 20,000 *E. maxima* oocysts on d 14 and 1 × 10^9^ *C. perfringens* cfu on d 18; T40K^+^, challenged with 40,000 *E. maxima* oocysts on d 14 and 1 × 10^9^ *C. perfringens* cfu on d 18.

2 L, linear effects; Q, quadratic effects.

3 dpi, days post inoculation of *E. maxima*.

**FIGURE 1:**
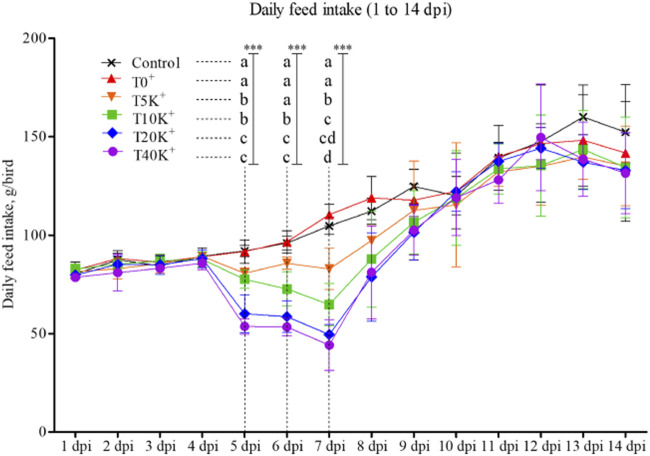
Daily feed intake trend in chickens infected with different doses of *E. maxima* oocysts and *C. perfringens* from 1 to 14 dpi (d 15–28). ^a–d^Means in each dpi with different superscripts indicate statistical differences (*p* < 0.001, denoted as ***). Each bar represents the standard error of the mean (*n* = 6). Abbreviations: dpi, days post inoculation of *E. maxima*; Control, non-challenged control; T0^+^, challenged with 1 × 10^9^ *C. perfringens* cfu on d 18; T5K^+^, challenged with 5,000 *E. maxima* oocysts on d 14 and 1 × 10^9^ *C. perfringens* cfu on d 18; T10K^+^, challenged with 10,000 *E. maxima* oocysts on d 14 and 1 × 10^9^ *C. perfringens* cfu on d 18; T20K^+^, challenged with 20,000 *E. maxima* oocysts on d 14 and 1 × 10^9^ *C. perfringens* cfu on d 18; T40K^+^, challenged with 40,000 *E. maxima* oocysts on d 14 and 1 × 10^9^ *C. perfringens* cfu on d 18.

The trend of daily feed intake (DFI) is presented in Figure 1. No significant difference in DFI was observed until 4 dpi, but a decrease in DFI was observed from 5 dpi onwards. On 5, 6, and 7 dpi, the T10K^+^, T20K^+^, and T40K^+^ groups showed decreased DFI compared to the Control and T0^+^ groups (*p* < 0.001). Additionally, even the lowest *E. maxima* oocyst dose group, T5K^+^, showed significantly lower DFI than the Control on 5 and 7 dpi. However, there were no statistical differences in DFI among the groups from 8 to 14 dpi. No statistical differences were observed between the Control group and the T0^+^ group throughout the entire period.

### 3.2 Jejunal NE lesion scores

**FIGURE 2:**
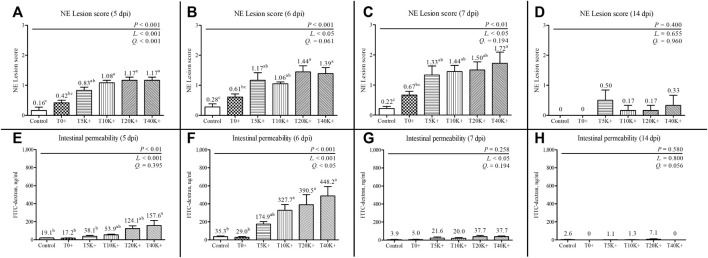
Jejunal necrotic enteritis lesion scores and intestinal permeability in chickens infected with different infection doses of *E. maxima* oocysts on 5, 6, 7, and 14 dpi (d 19, 20, 21, and 28). Bars with different superscripts (a, b, and c**)** indicate statistical differences (*p* < 0.05). Each bar represents the standard error of the mean (*n* = 6). Abbreviations: dpi, days post inoculation of *E. maxima*; Control, non-challenged control; T0^+^, challenged with 1 × 10^9^ *C. perfringens* cfu on d 18; T5K^+^, challenged with 5,000 *E. maxima* oocysts on d 14 and 1 × 10^9^ *C. perfringens* cfu on d 18; T10K^+^, challenged with 10,000 *E. maxima* oocysts on d 14 and 1 × 10^9^ *C. perfringens* cfu on d 18; T20K^+^, challenged with 20,000 *E. maxima* oocysts on d 14 and 1 × 10^9^ *C. perfringens* cfu on d 18; T40K^+^, challenged with 40,000 *E. maxima* oocysts on d 14 and 1 × 10^9^ *C. perfringens* cfu on d 18; L, linear effects; Q, quadratic effects. **(A)** NE lesion scores on 5 dpi. **(B)** NE lesion scores on 6 dpi. **(C)** NE lesion scores on 7 dpi. **(D)** NE lesion scores on 14 dpi. **(E)** Intestinal permeability on 5 dpi. **(F)** Intestinal permeability on 6 dpi. **(G)** Intestinal permeability on 7 dpi. **(H)** Intestinal permeability on 14 dpi.

The results of jejunal NE lesion scores are shown in Figure 2. The NE lesion scores exhibited a significant increase with increasing levels of *E. maxima* oocyst dose in combination with *C. perfringens* coinfection at 5 dpi (linear and quadratic, *p* < 0.001), 6 dpi (linear, *p* < 0.05), and 7 dpi (linear, *p* < 0.05). The T5K^+^, T10K^+^, T20K^+^, and T40K^+^ groups showed increased jejunal NE lesion scores compared to the Control group on 5, 6, and 7 dpi (5 dpi, *p* < 0.001; 6 dpi, *p* < 0.001; 7 dpi, *p* < 0.01). The T0^+^ group had significantly lower NE lesion scores than the T10K^+^, T20K^+^, or T40K^+^ groups on 5, 6, or 7 dpi. However, no significant difference in NE lesion scores was observed between the Control and T0^+^ groups at any of the sampling time points (5, 6, 7, and 14 dpi), indicating that *C. perfringens* challenge alone is insufficient to induce NE in broiler chickens. There were no significant differences in NE lesion scores among the treatment groups at 14 dpi.

### 3.3 Intestinal permeability

The results of intestinal permeability are presented in Figure 2. Intestinal permeability significantly increased with increasing levels of *E. maxima* oocyst doses with coinfection of *C. perfringens* at 5 dpi (linear, *p* < 0.001), 6 dpi (linear and quadratic, *p* < 0.001 and *p* < 0.05), and 7 dpi (linear, *p* < 0.05). On 5 dpi, the T40K^+^ group showed increased intestinal permeability compared to the Control and T0^+^ groups (*p* < 0.01). At 6 dpi, the T10K^+^, T20K^+^, and T40K^+^ groups showed increased intestinal permeability in comparison to the Control and T0^+^ groups (*p* < 0.001). No significant difference in intestinal permeability was observed between the Control and T0^+^ groups at any of the sampling time points (5, 6, 7, and 14 dpi). There were no statistical differences in intestinal permeability among all groups at 7 and 14 dpi.

### 3.4 Jejunal *C. perfringens* colony counting

**FIGURE 3:**
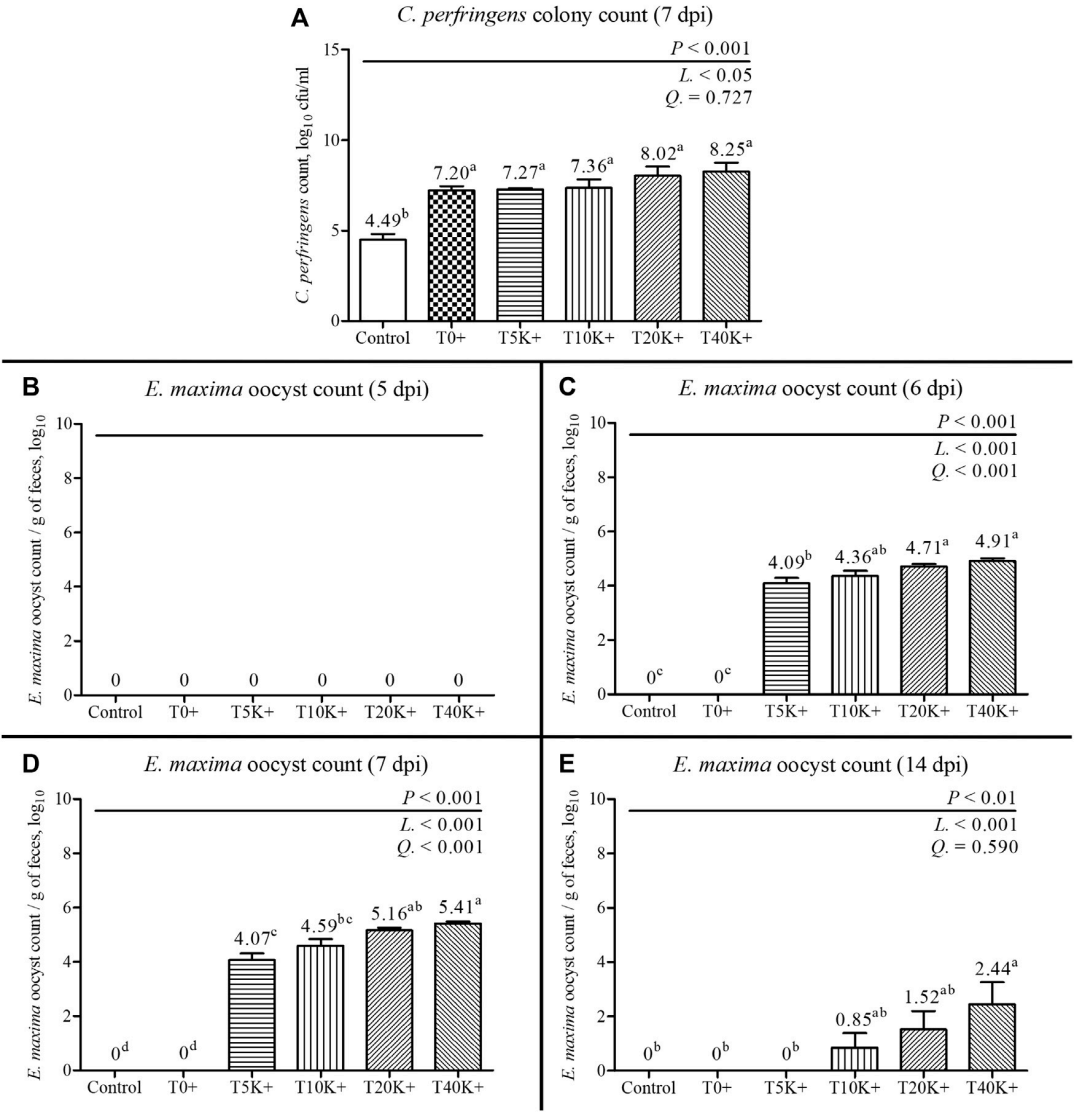
*C. perfringens* colony count of jejunal digesta and fecal *E. maxima* oocyst counts in chickens infected with different doses of *E. maxima* oocysts and *C. perfringens* on 5, 6, 7, and 14 dpi (d 19, 20, 21, and 28). Bars with different superscripts (a, b, c, and d indicate statistical differences (*p* < 0.05). Each bar represents the standard error of the mean (*n* = 6). Abbreviations: dpi, days post inoculation of *E. maxima*; Control, non-challenged control; T0^+^, challenged with 1 × 10^9^ *C. perfringens* cfu on d 18; T5K^+^, challenged with 5,000 *E. maxima* oocysts on d 14 and 1 × 10^9^ *C. perfringens* cfu on d 18; T10K^+^, challenged with 10,000 *E. maxima* oocysts on d 14 and 1 × 10^9^ *C. perfringens* cfu on d 18; T20K^+^, challenged with 20,000 *E. maxima* oocysts on d 14 and 1 × 10^9^ *C. perfringens* cfu on d 18; T40K^+^, challenged with 40,000 *E. maxima* oocysts on d 14 and 1 × 10^9^ *C. perfringens* cfu on d 18; L, linear effects; Q, quadratic effects. **(A)** Jejunal *C. perfringens* colony count on 7 dpi. **(B)** Fecal *E. maxima* oocyst count on 5 dpi. **(C)** Fecal *E. maxima* oocyst count on 6 dpi. **(D)** Fecal *E. maxima* oocyst count on 7 dpi. **(E)** Fecal *E. maxima* oocyst count on 14 dpi.

The result of jejunal *C. perfringens* colony count is shown in Figure 3. There was a significant increase in *C. perfringens* colony count with increasing levels of *E. maxima* oocyst doses with *C. perfringens* coinfection at 7 dpi (linear, *p* < 0.05), indicating that the dose of *E. maxima* inoculation plays a crucial role in the colonization of *C. perfringens* in the dual-infection NE model. All groups inoculated with *C. perfringens* (T0^+^ to T40K^+^) exhibited higher jejunal *C. perfringens* colony counts compared to the Control group (*p* < 0.001). There was no statistical difference among the groups challenged with *C. perfringens*.

### 3.5 Fecal *E. maxima* oocyst counting

The results of fecal *E. maxima* oocyst count are presented in Figure 3. In the Control and T0^+^ groups, no *E. maxima* oocysts were detected at any sampling time point. On 5 dpi, no *E. maxima* oocysts were observed in any of the groups. However, on 6, 7, and 14 dpi, the fecal *E. maxima* oocyst counts significantly increased with increasing levels of *E. maxima* oocyst dose with coinfection of *C. perfringens* (6 and 7 dpi, linear and quadratic, *p* < 0.001; 14 dpi, linear, *p* < 0.001). The T20K^+^ and T40K^+^ groups showed increased fecal *E. maxima* oocysts compared to the Control, T0^+^, and T5K^+^ groups at 6 and 7 dpi (*p* < 0.001). On 14 dpi, *E. maxima* oocysts were observed in the T10K^+^, T20K^+^, and T40K^+^ groups, with the T40K^+^ group showed higher *E. maxima* oocysts compared to the Control, T0^+^, and T5K^+^ groups (*p* < 0.01).

### 3.6 Apparent ileal digestibility and water content

**FIGURE 4:**
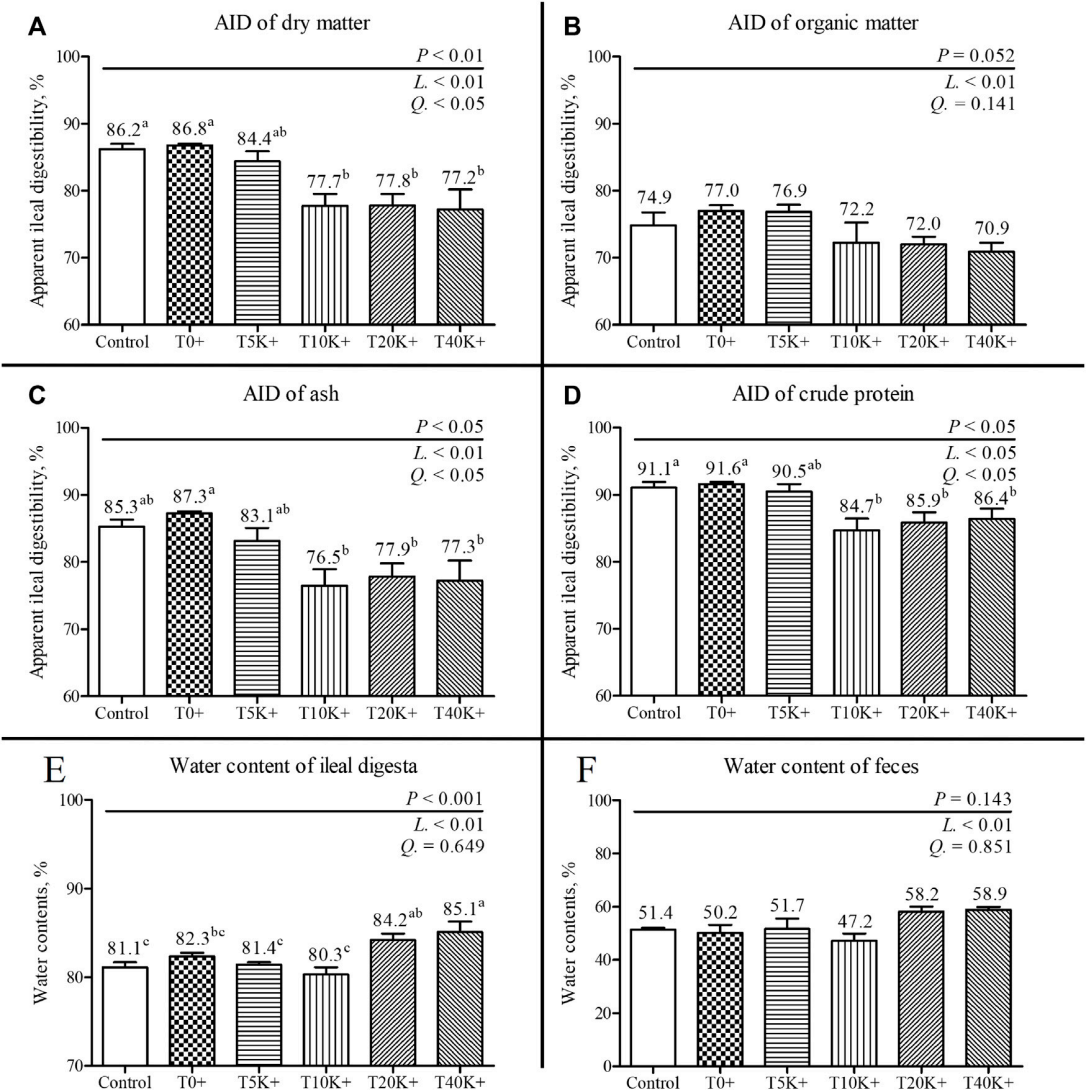
Apparent ileal digestibility (AID) and water content of ileal digesta and feces in chickens infected with different doses of *E. maxima* oocysts and *C. perfringens* on 7 dpi (d 21). Bars with different superscripts (a, b, and c indicate statistical differences (*p* < 0.05). Each bar represents the standard error of the mean (n = 6). Abbreviations: Control, non-challenged control; T0^+^, challenged with 1 × 10^9^ *C. perfringens* cfu on d 18; T5K^+^, challenged with 5,000 *E. maxima* oocysts on d 14 and 1 × 10^9^ *C. perfringens* cfu on d 18; T10K^+^, challenged with 10,000 *E. maxima* oocysts on d 14 and 1 × 10^9^ *C. perfringens* cfu on d 18; T20K^+^, challenged with 20,000 *E. maxima* oocysts on d 14 and 1 × 10^9^ *C. perfringens* cfu on d 18; T40K^+^, challenged with 40,000 *E. maxima* oocysts on d 14 and 1 × 10^9^ *C. perfringens* cfu on d 18; L, linear effects; Q, quadratic effects. **(A)** Dry matter AID on 7 dpi. **(B)** Organic matter AID on 7 dpi. **(C)** Ash AID on 7 dpi. **(D)** Crude protein AID on 14 dpi. **(E)** Water content of ileal digesta on 7 dpi. **(F)** Water content of feces on 7 dpi.

The results of AID and the water content of the ileal digesta and feces are shown in Figure 4. On 7 dpi, all parameters of AID (DM, OM, crude protein, and ash) exhibited a significant decrease with increasing levels of *E. maxima* with *C. perfringens* coinfection (DM, linear and quadratic, *p* < 0.01 and *p* < 0.05; OM, linear, *p* < 0.01; ash, linear and quadratic, *p* < 0.01 and *p* < 0.05; crude protein, linear and quadratic, *p* < 0.05). The T10K^+^, T20K^+^, and T40K^+^ groups showed reduced AID of DM and crude protein compared to the Control and T0^+^ groups (DM, *p* < 0.01; crude protein, *p* < 0.05). However, there was no significant difference in AID of OM among the treatment groups. The T10K^+^, T20K^+^, and T40K^+^ groups showed decreased AID of ash compared to the T0^+^ group (*p* < 0.05). The water content of the ileal digesta and feces significantly increased with increasing levels of *E. maxima* oocyst dose with coinfection of *C. perfringens* at 7 dpi (linear, *p* < 0.01). The T20K^+^ and T40K^+^ groups showed significantly increased water content compared to the Control, T5K^+^, and T10K^+^ groups, with only the T40K^+^ group showing a significant difference compared to the T0^+^ group. There were no statistical differences between the Control group and the T0^+^ group in terms of all AID parameters and water content.

### 3.7 Intestinal morphology

**FIGURE 5:**
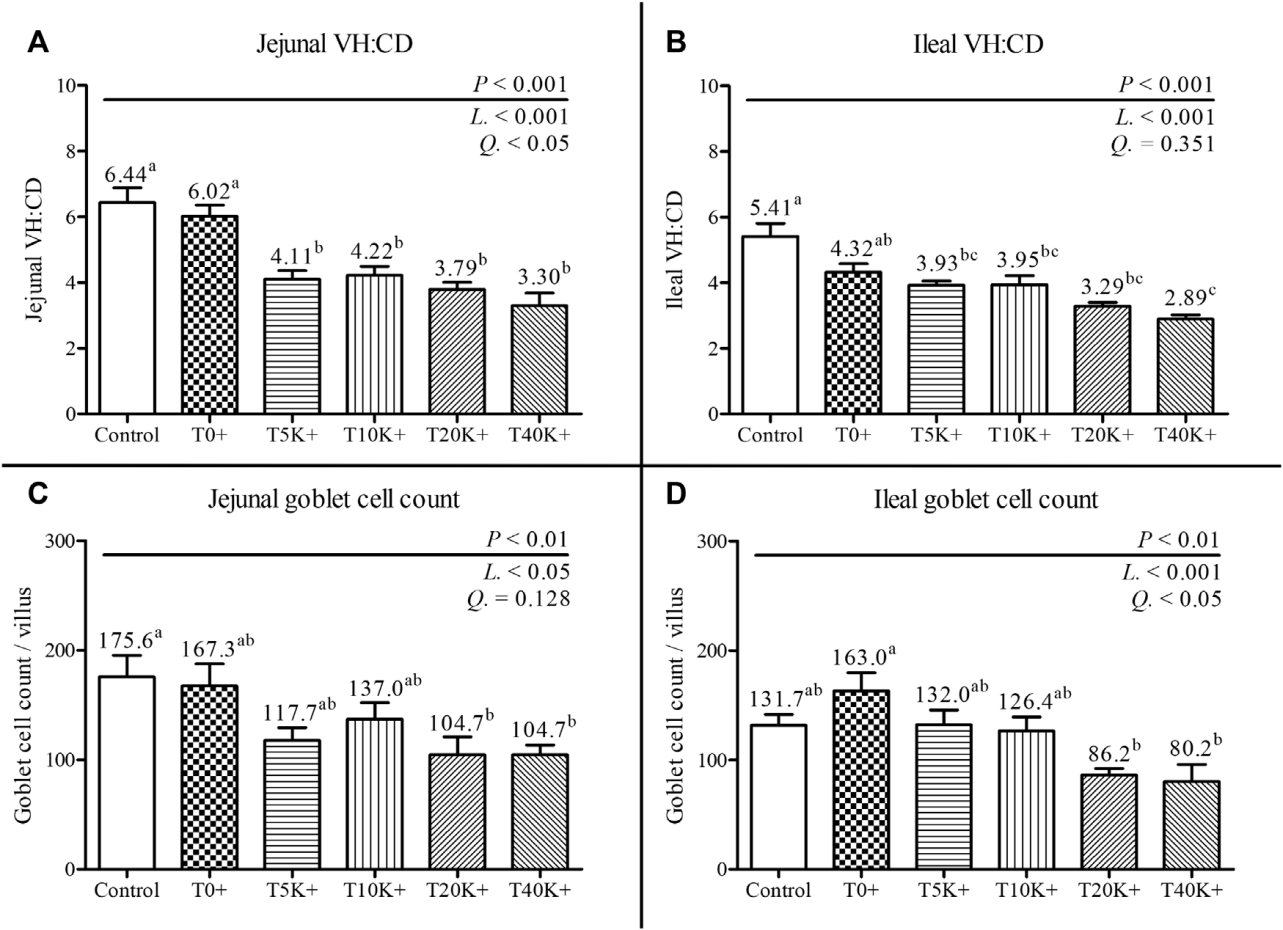
Jejunal and ileal morphology in chickens infected with different doses of *E. maxima* oocysts and *C. perfringens* on 6 dpi (d 20). Bars with different superscripts (a, b, and c indicate statistical differences (*p* < 0.05). Each bar represents the standard error of the mean (*n* = 6). Abbreviations: Control, non-challenged control; T0^+^, challenged with 1 × 10^9^ *C. perfringens* cfu on d 18; T5K^+^, challenged with 5,000 *E. maxima* oocysts on d 14 and 1 × 10^9^ *C. perfringens* cfu on d 18; T10K^+^, challenged with 10,000 *E. maxima* oocysts on d 14 and 1 × 10^9^ *C. perfringens* cfu on d 18; T20K^+^, challenged with 20,000 *E. maxima* oocysts on d 14 and 1 × 10^9^ *C. perfringens* cfu on d 18; T40K^+^, challenged with 40,000 *E. maxima* oocysts on d 14 and 1 × 10^9^ *C. perfringens* cfu on d 18; VH:CD, villus height to crypt depth ratio; L, linear effects; Q, quadratic effects. **(A)** Jejunal VH:CD on 6 dpi. **(B)** Ileal VH:CD on 6 dpi. **(C)** Jejunal goblet cell count on 6 dpi. **(D)** Ileal goblet cell count on 6 dpi.

**FIGURE 6:**
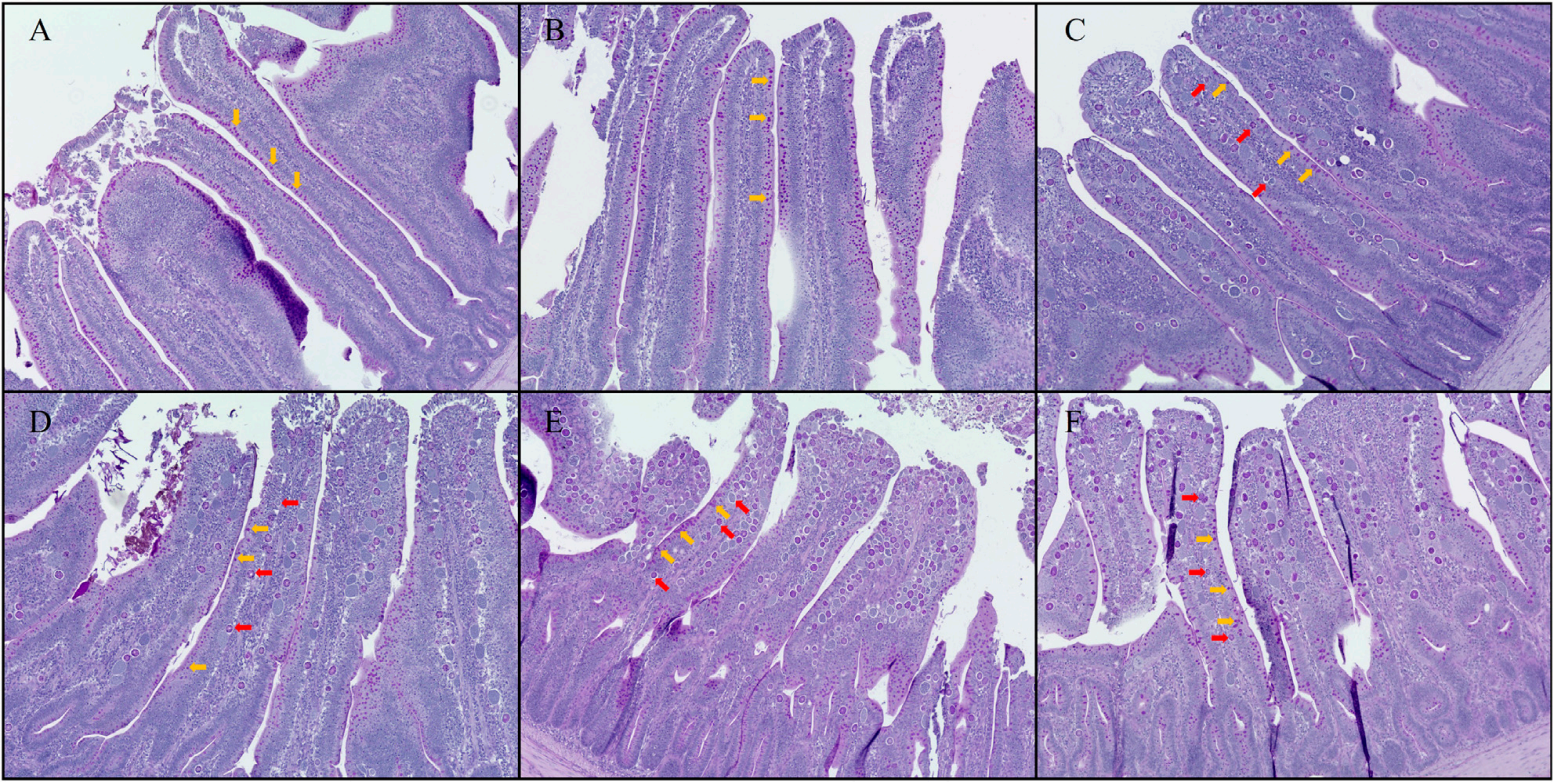
Hematoxylin counterstained Period acid-Schiff (PAS) stained jejunal morphology in chickens infected with different doses of *E. maxima* oocysts and *C. perfringens* on 6 dpi (d 20). **(A)** Control, non-challenged control. **(B)** T0^+^, challenged with 1 × 10^9^ *C. perfringens* cfu on d 18. **(C)** T5K^+^, challenged with 5,000 *E. maxima* oocysts on d 14 and 1 × 10^9^ *C. perfringens* cfu on d 18. **(D)** T10K^+^, challenged with 10,000 *E. maxima* oocysts on d 14 and 1 × 10^9^ *C. perfringens* cfu on d 18. **(E)** T20K^+^, challenged with 20,000 *E. maxima* oocysts on d 14 and 1 × 10^9^ *C. perfringens* cfu on d 18. **(F)** T40K^+^, challenged with 40,000 *E. maxima* oocysts on d 14 and 1 × 10^9^ *C. perfringens* cfu on d 18. The orange arrows point to goblet cells, and the red arrows point to *E. maxima*. Based on the magnification of **(A,B)**, group **(C–F)** were enlarged by 1.25 times.

The results of jejunal and ileal VH:CD and GC count are presented in Figure 5. Additionally, representative jejunal morphology images of the groups are shown in Figure 6. Increasing levels of *E. maxima* with coinfection of *C. perfringens* significantly decreased jejunal and ileal VH:CD and villus GC counts at 6 dpi (jejunal VH:CD, linear and quadratic, *p* < 0.001 and *p* < 0.05; ileal VH:CD, linear, *p* < 0.001; jejunal GC count, linear, *p* < 0.05; ileal GC count, linear and quadratic, *p* < 0.001 and *p* < 0.05). The T5K^+^, T10K^+^, T20K^+^, and T40K^+^ groups showed decreased jejunal VH:CD compared to the Control and T0^+^ groups (*p* < 0.001). The T40K^+^ group had decreased ileal VH:CD compared to the Control and T0^+^ groups (*p* < 0.001), and the T5K^+^, T10K^+^, and T20K^+^ groups had significantly lower ileal VH:CD than the Control group. In terms of GC count, the T20K^+^ and T40K^+^ groups showed decreased jejunal GC count compared to the Control group (*p* < 0.01), and the T20K^+^ and T40K^+^ groups had decreased ileal GC count compared to the T0^+^ group (*p* < 0.01). There were no statistical differences in VH:CD and GC count between the Control group and the T0^+^ group. This suggests that *C. perfringens* alone may not be sufficient to cause severe changes in intestinal morphology and function in broilers.

### 3.8 Jejunal gene expression

The results of relative jejunal gene expression are presented in Table 5. Increasing levels of *E. maxima* with *C. perfringens* coinfection led to significant decrease of expression of some mRNAs, including mucin 2 (MUC2; linear and quadratic *p* < 0.001), sodium-dependent amino acid transporter (B^0^AT; linear, *p* < 0.05), sodium-independent amino acid transporter (B^0,+^AT; linear and quadratic, *p* < 0.01), peptide transporter 1 (PepT1; linear and quadratic, *p* < 0.001 and *p* < 0.05), glucose transporter 2 (GLUT2; linear and quadratic, *p* < 0.01), toll-like receptor 4 (TLR4; linear and quadratic, *p* < 0.01 and *p* < 0.05), avian beta-defensin 3 (AvBD3; linear, *p* < 0.05), avian beta-defensin 9 (AvBD9; linear, *p* < 0.05), liver-expressed antimicrobial peptides 2 (LEAP2; linear and quadratic, *p* < 0.01 and *p* < 0.001). Additionally, there was an upregulation of claudin 1 (CLDN1; linear, *p* < 0.05), interleukin 1 beta (IL-1β; linear, *p* < 0.01), interferon gamma (IFN-γ; linear, *p* < 0.01), tumor necrosis factor superfamily 15 (TNFSF15; linear, *p* < 0.05), TNF-α (linear, *p* < 0.05), IL-10 (linear, *p* < 0.05), cathelicidin 3 (CATHL3; linear, *p* < 0.05), *E. maxima* gametocyte 56 kDa (Gam56; linear, *p* < 0.05), and Gam82 (linear and quadratic, *p* < 0.05). Increasing levels of *E. maxima* oocyst dose with *C. perfringens* coinfection quadratically changed occludin (OCLN; quadratic, *p* < 0.05), zonula occludens 2 (ZO2; quadratic, *p* < 0.001), and excitatory amino acid transporter 3 (EAAT3; quadratic, *p* < 0.01) gene expressions. Furthermore, the T10K^+^, T20K^+^, and T40K^+^ groups showed significant differences in gene expression compared to the Control group. Specifically, CLDN1 was upregulated (*p* < 0.001), MUC2 was downregulated (*p* < 0.05), B^0,+^AT was downregulated (*p* < 0.001), IL-2 was downregulated (*p* < 0.001), and AvBD9 was downregulated (*p* < 0.001) in these groups. The T40K^+^ group also showed upregulation of INF-γ compared to the Control and T0^+^ groups (*p* < 0.05). In addition, the T20K^+^ group exhibited upregulation of IL-1β (*p* < 0.01) and downregulation of LEAP2 (*p* < 0.001) compared to the Control and T0^+^ groups. Among the groups challenged with *E. maxima* (T5K^+^, T10K^+^, T20K^+^, and T40K^+^), the T20K^+^ group showed upregulation of Gam82 compared to the T5K^+^ group (*p* < 0.05). However, there were no differences in gene expression levels observed between the T20K^+^ and T40K^+^ groups or between the T5K^+^ and T10K^+^ groups in any of the categories.

**TABLE 5:** Effects of different doses of *E. maxima* oocysts with *C. perfringens* on the relative jejunal gene expression in broiler chickens on d 20 (6 dpi).

	Treatments^1^						SEM (*n* = 6)	*P*-value^2^		
	Control	T0^+^	T5K^+^	T10K^+^	T20K^+^	T40K^+^		ANOVA	L	Q
Tight junction proteins										
CLDN1	1.00^b^	0.69^b^	3.29^a^	3.58^a^	2.97^a^	3.79^a^	0.445	<0.001	<0.05	0.059
JAM2	1.00	1.17	0.83	0.68	0.75	0.70	0.167	0.164	0.135	0.116
OCLN	1.00	1.56	1.09	0.96	0.80	1.07	0.221	0.169	0.211	<0.05
ZO2	1.00	0.86	0.54	0.52	0.42	0.62	0.186	0.102	0.164	<0.001
Mucin										
MUC2	1.00^a^	0.68^ab^	0.38^ab^	0.29^b^	0.25^b^	0.28^b^	0.194	<0.05	<0.001	<0.001
Nutrient transporters										
B^0^AT	1.00	0.68	0.53	0.50	0.40	0.39	0.259	0.441	<0.05	0.090
B^0,+^AT	1.00^a^	0.96^a^	0.50^ab^	0.41^b^	0.26^b^	0.29^b^	0.143	<0.001	<0.01	<0.01
EAAT3	1.00	0.56	0.36	0.37	0.31	0.40	0.187	0.058	0.234	<0.01
PepT1	1.00	0.72	0.51	0.38	0.31	0.23	0.281	0.243	<0.001	<0.05
GLUT2	1.00^b^	1.98^a^	0.84^b^	0.73^b^	0.61^b^	0.63^b^	0.230	<0.01	<0.01	<0.01
Host defense peptides										
AvBD3	1.00	0.60	0.45	0.21	0.19	0.08	0.244	0.117	<0.05	0.156
AvBD9	1.00^a^	0.52^ab^	0.48^ab^	0.27^b^	0.27^b^	0.20^b^	0.138	<0.001	<0.05	0.266
CATHL3	1.00	1.00	2.55	2.41	3.08	3.23	0.726	0.071	<0.05	0.194
LEAP2	1.00^ab^	1.45^a^	0.59^bc^	0.35^bc^	0.28^c^	0.37^bc^	0.176	<0.001	<0.01	<0.001
TLR/NF-κB signaling pathway-related genes										
NF-κB	1.00	0.83	0.70	0.99	1.36	0.95	0.357	0.767	0.515	0.282
TLR2	1.00	0.83	0.52	0.48	0.81	0.67	0.326	0.833	0.932	0.850
TLR4	1.00	0.86	0.79	0.63	0.53	0.51	0.127	0.054	<0.01	<0.05
Cytokines										
IL-1β	1.00^b^	1.16^b^	1.13^b^	1.10^b^	2.06^a^	1.69^ab^	0.184	<0.01	<0.01	0.150
IL-2	1.00^a^	0.53^ab^	0.26^b^	0.20^b^	0.30^b^	0.17^b^	0.134	<0.001	0.058	0.327
IFN-γ	1.00^b^	1.18^b^	3.36^ab^	3.37^ab^	3.85^ab^	5.40^a^	0.904	<0.05	<0.01	0.426
TNFSF15	1.00	0.58	0.83	1.42	2.27	2.11	0.522	0.106	<0.05	0.154
* *TNF-α	1.00	0.67	0.65	0.69	0.96	0.95	0.126	0.123	<0.05	0.459
IL-6	1.00	0.99	0.67	0.62	0.99	0.72	0.376	0.928	0.885	0.998
IL-10	1.00^ab^	0.59^b^	1.49^ab^	1.64^ab^	1.72^ab^	2.13^a^	0.357	<0.05	<0.05	0.225
*E. maxima* gametocytes										
APN	—	—	1.00	1.32	2.02	1.33	0.260	0.051	0.139	0.064
EF2	—	—	1.00	1.18	1.39	1.45	0.187	0.292	0.055	0.727
Gam56	—	—	1.00	1.47	1.99	1.82	0.289	0.097	<0.05	0.228
Gam82	—	—	1.00^b^	1.59^ab^	2.14^a^	1.67^ab^	0.255	<0.05	<0.05	<0.05
IMC1	—	—	1.00	1.52	1.52	1.36	0.568	0.886	0.655	0.496

^a–c^Means in the same row with different superscripts indicate statistically differences (*P* < 0.05).

1 Control, non-challenged control; T0^+^, challenged with 1 × 10^9^ *C. perfringens* cfu on d 18; T5K^+^, challenged with 5,000 *E. maxima* oocysts on d 14 and 1 × 10^9^ *C. perfringens* cfu on d 18; T10K^+^, challenged with 10,000 *E. maxima* oocysts on d 14 and 1 × 10^9^ *C. perfringens* cfu on d 18; T20K^+^, challenged with 20,000 *E. maxima* oocysts on d 14 and 1 × 10^9^ *C. perfringens* cfu on d 18; T40K^+^, challenged with 40,000 *E. maxima* oocysts on d 14 and 1 × 10^9^ *C. perfringens* cfu on d 18.

2 L, linear effects; Q, quadratic effects.

Abbreviations: CLDN1, claudin 1; JAM2, junctional adhesion molecule 2; OCLN, occludin; ZO2, zonula occludens 2; MUC2, mucin 2; B^0^AT, sodium dependent amino acid transporter (solute carrier family 6 member 19, SLC6A19); B^0,+^AT, sodium independent amino acid transporter (solute carrier family 7 member 9, SLC7A9); EAAT3, excitatory amino acid transporter 3 (solute carrier family 1 member 1, SLC1A1); PepT1, peptide transporter 1 (solute carrier family 15 member 1, SLC15A1); GLUT2, glucose transporter 2 (solute carrier family 2 member 2, SLC2A2); AvBD, avian beta-defensin; CATHL3, cathelicidin 3; LEAP2, liver-expressed antimicrobial peptides 2; NF-κB, nuclear factor kappa B p 65; TLR, toll-like receptor; IL, interleukin; IFN-γ, interferon gamma; TNFSF15, tumor necrosis factor superfamily 15; TNF-α, tumor necrosis factor alpha; APN, *E. maxima* aminopeptidase N; EF2, elongation factor 2; Gam, *E. maxima* gametocyte antigen; IMC1, membrane skeletal protein inner membrane complex 1.

### 3.9 Alpha-diversity of cecal bacterial communities

**FIGURE 7:**
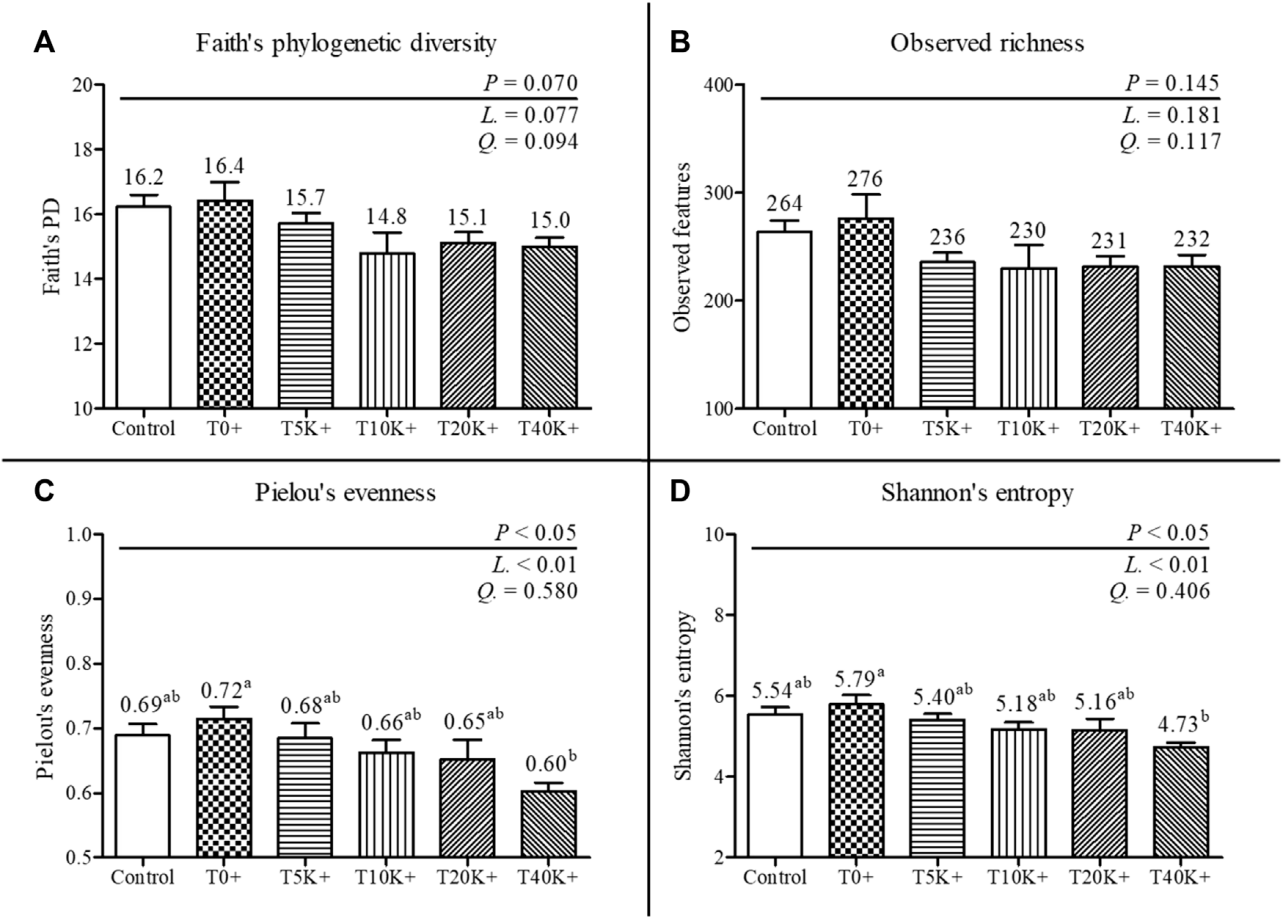
Alpha-diversity of cecal bacterial communities in chickens infected with different doses of *E. maxima* oocysts and *C. perfringens* on 6 dpi (d 20). Bars with different superscripts (a and b) indicate statistical differences (*p* < 0.05). Each bar represents the standard error of the mean (*n* = 6). Abbreviations: Control, non-challenged control; T0^+^, challenged with 1 × 10^9^ *C. perfringens* cfu on d 18; T5K^+^, challenged with 5,000 *E. maxima* oocysts on d 14 and 1 × 10^9^ *C. perfringens* cfu on d 18; T10K^+^, challenged with 10,000 *E. maxima* oocysts on d 14 and 1 × 10^9^ *C. perfringens* cfu on d 18; T20K^+^, challenged with 20,000 *E. maxima* oocysts on d 14 and 1 × 10^9^ *C. perfringens* cfu on d 18; T40K^+^, challenged with 40,000 *E. maxima* oocysts on d 14 and 1 × 10^9^ *C. perfringens* cfu on d 18; L, linear effects; Q, quadratic effects. **(A)** Faith’s phylogenetic diversity on 6 dpi. **(B)** Observed richness on 6 dpi. **(C)** Pielou’s evenness on 6 dpi. **(D)** Shannon’s entropy on 6 dpi.

The alpha-diversity results of cecal bacterial communities are presented in Figure 7. The total number of features identified was 1,195, with a minimum frequency of 30,450 and a maximum of 66,819. No statistical differences were observed between the Control group and the T0^+^ group in any of the alpha-diversity parameters. Increasing levels of *E. maxima* with *C. perfringens* coinfection led to a significant decrease in Pielou’s evenness and Shannon’s entropy of cecal bacterial communities at 6 dpi (linear, *p* < 0.01). No differences were found in Faith’s phylogenetic diversity and Observed richness among the groups. The T40K^+^ group showed decreased Pielou’s evenness and Shannon’s entropy compared to the T0^+^ group (*p* < 0.05).

### 3.10 Beta-diversity of cecal bacterial communities

**FIGURE 8:**
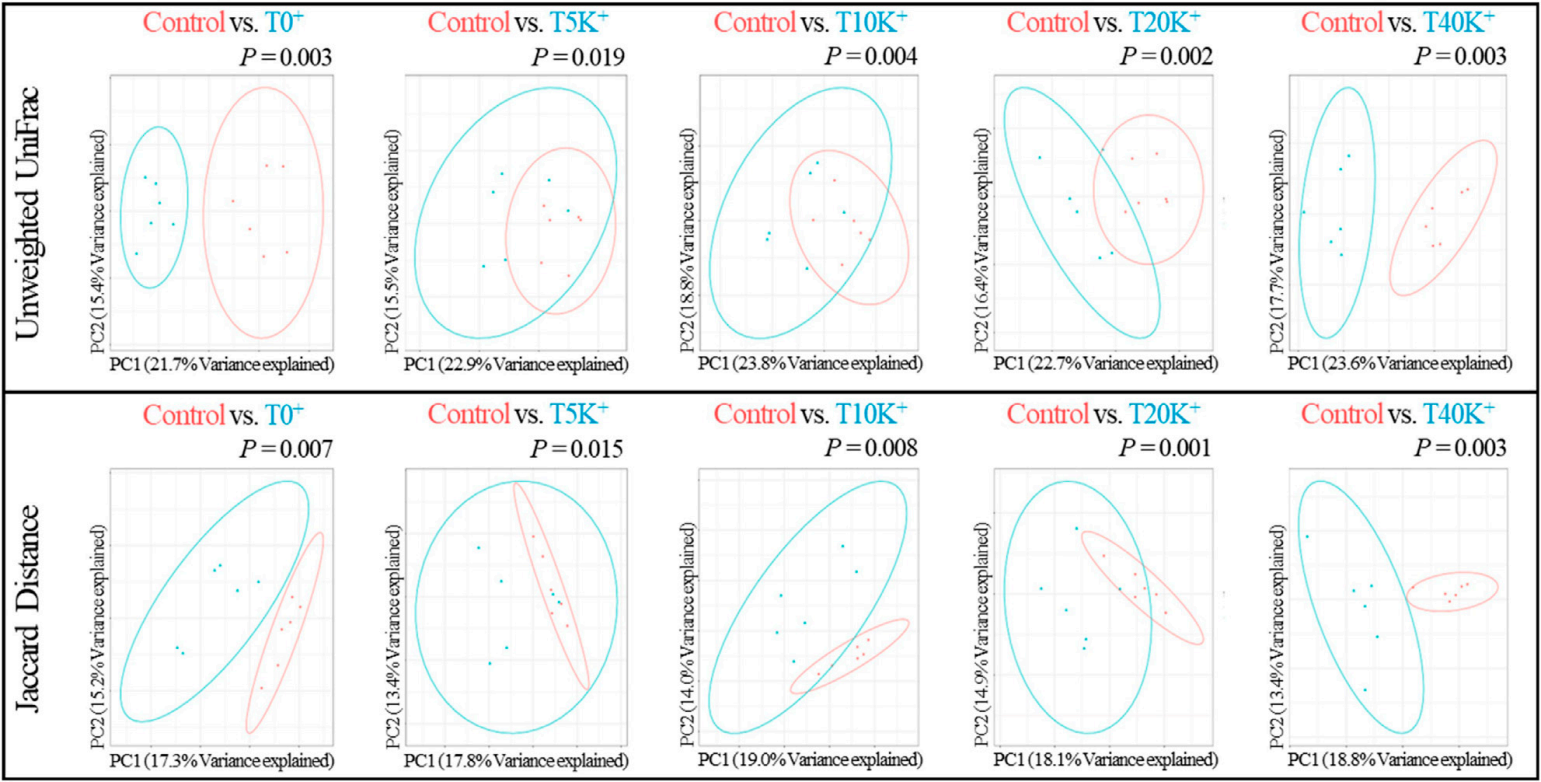
The principal coordinate analysis (PCoA) plots based on Unweighted UniFrac and Jaccard distance of cecal bacterial communities in chickens infected with different doses of *E. maxima* oocysts and *C. perfringens* on 6 dpi (d 20). Differences were assessed using permutation multivariate analysis of variance (PERMANOVA) with 999 permutations. Abbreviations: Control, non-challenged control; T0^+^, challenged with 1 × 10^9^ *C. perfringens* cfu on d 18; T5K^+^, challenged with 5,000 *E. maxima* oocysts on d 14 and 1 × 10^9^ *C. perfringens* cfu on d 18; T10K^+^, challenged with 10,000 *E. maxima* oocysts on d 14 and 1 × 10^9^ *C. perfringens* cfu on d 18; T20K^+^, challenged with 20,000 *E. maxima* oocysts on d 14 and 1 × 10^9^ *C. perfringens* cfu on d 18; T40K^+^, challenged with 40,000 *E. maxima* oocysts on d 14 and 1 × 10^9^ *C. perfringens* cfu on d 18; PC, principal coordinate.

The results of beta-diversity analysis using Unweighted UniFrac and Jaccard distance for cecal bacterial communities are presented in Figure 8. PCoA plots were generated to compare the differences between the Control group and the challenged groups (T0^+^, T5K^+^, T10K^+^, T20K^+^, and T40K^+^) and assessed the dissimilarity of cecal bacterial communities at 6 dpi using PERMANOVA. Based on the Weighted UniFrac distances and Bray-Curtis dissimilarity measures, no statistical differences were observed in the PCoA plots between the challenged groups and the Control group (data not shown). However, in the PCoA plots based on Unweighted UniFrac distances, all challenged groups showed significant differences compared to the Control group (T0^+^, *p* < 0.01; T5K^+^, *p* < 0.05; T10K^+^, *p* < 0.01; T20K^+^, *p* < 0.01; T40K^+^, *p* < 0.01). Similarly, in the PCoA plots based on Jaccard distance, all challenged groups showed significant differences compared to the Control group (T0^+^, *p* < 0.01; T5K^+^, *p* < 0.05; T10K^+^, *p* < 0.01; T20K^+^, *p* < 0.01; T40K^+^, *p* < 0.01).

### 3.11 Taxonomic compositions of cecal bacterial communities

**FIGURE 9:**
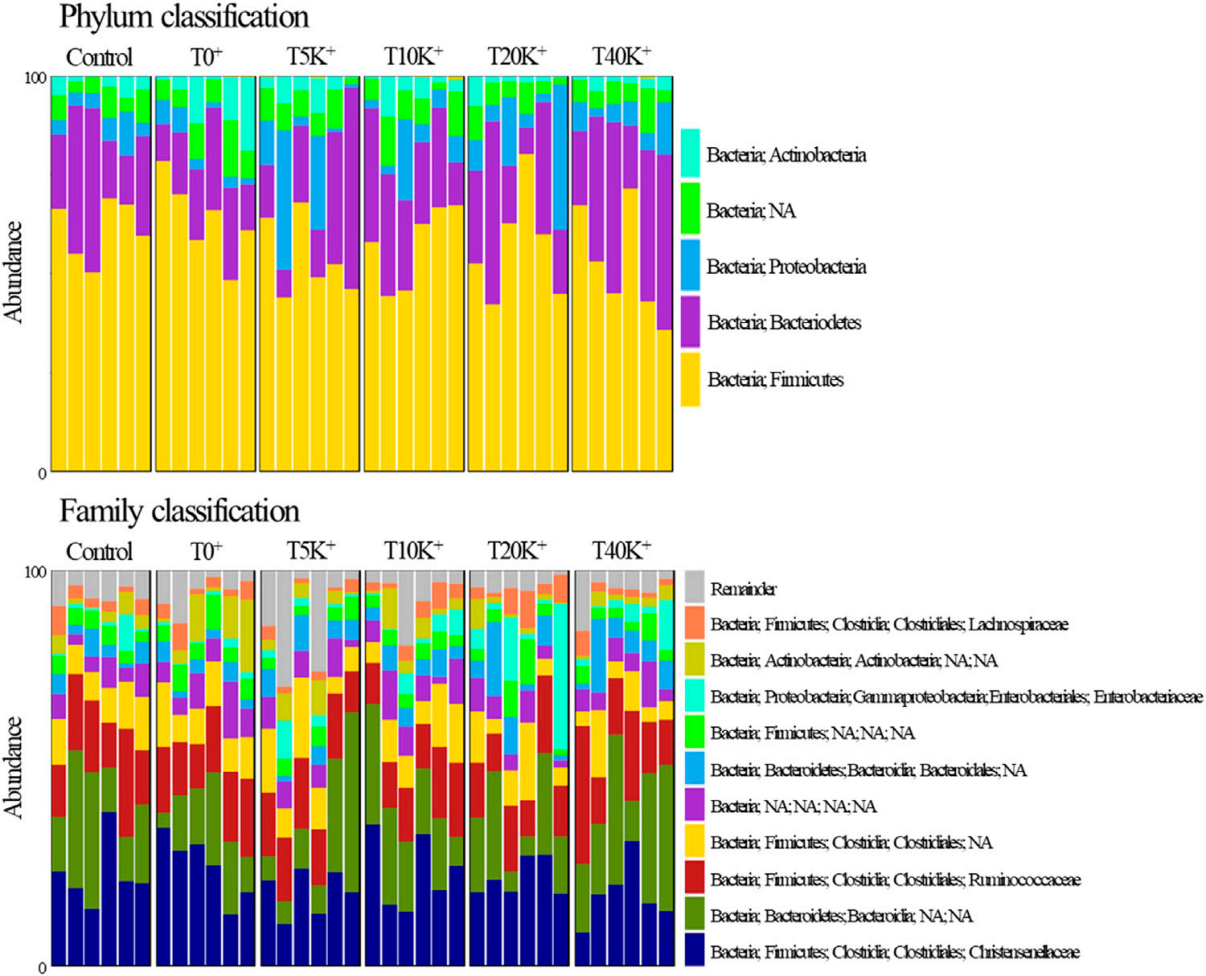
The composition of phylum- and family-level cecal bacterial communities in chickens infected with different doses of *E. maxima* oocysts and *C. perfringens* on 6 dpi (d 20). Abbreviations: Control, non-challenged control; T0^+^, challenged with 1 × 10^9^ *C. perfringens* cfu on d 18; T5K^+^, challenged with 5,000 *E. maxima* oocysts on d 14 and 1 × 10^9^ *C. perfringens* cfu on d 18; T10K^+^, challenged with 10,000 *E. maxima* oocysts on d 14 and 1 × 10^9^ *C. perfringens* cfu on d 18; T20K^+^, challenged with 20,000 *E. maxima* oocysts on d 14 and 1 × 10^9^ *C. perfringens* cfu on d 18; T40K^+^, challenged with 40,000 *E. maxima* oocysts on d 14 and 1 × 10^9^ *C. perfringens* cfu on d 18; NA, non-assigned.

**FIGURE 10:**
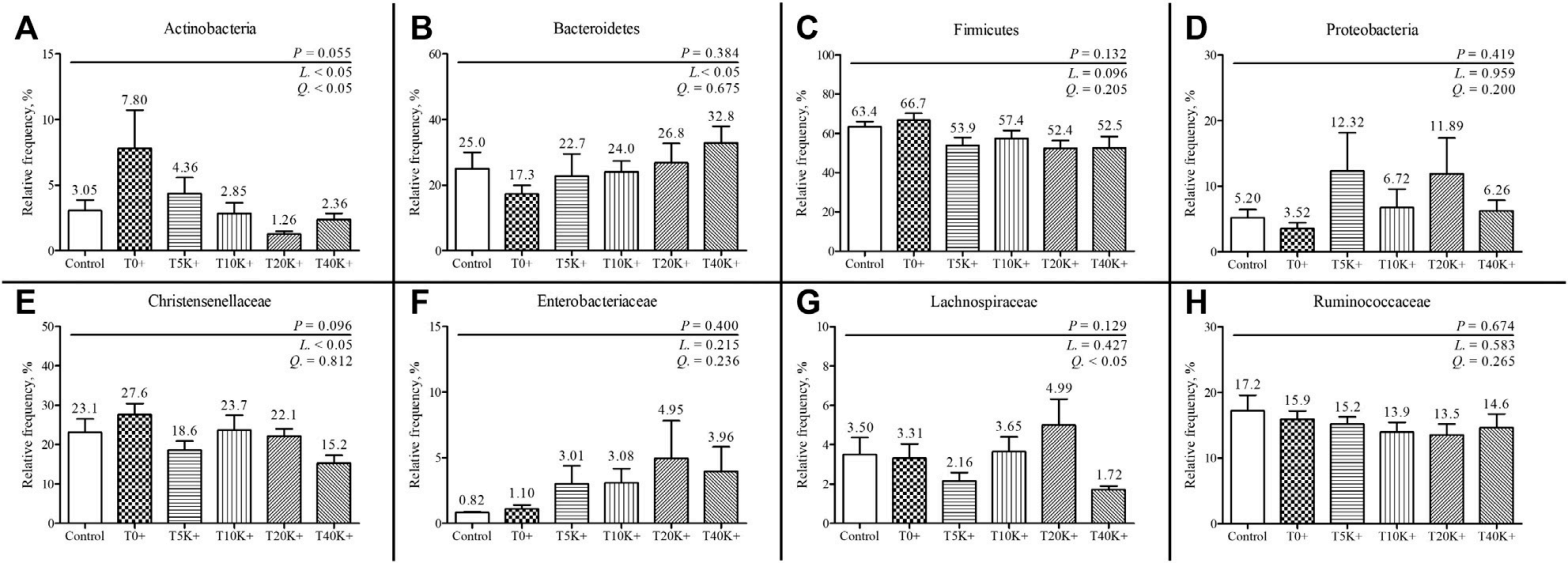
The relative frequency of phylum and family-level composition in cecal bacterial communities in chickens infected with different doses of *E. maxima* oocysts and *C. perfringens* on 6 dpi (d 20). Each bar represents the standard error of the mean (*n* = 6). Abbreviations: Control, non-challenged control; T0^+^, challenged with 1 × 10^9^ *C. perfringens* cfu on d 18; T5K^+^, challenged with 5,000 *E. maxima* oocysts on d 14 and 1 × 10^9^ *C. perfringens* cfu on d 18; T10K^+^, challenged with 10,000 *E. maxima* oocysts on d 14 and 1 × 10^9^ *C. perfringens* cfu on d 18; T20K^+^, challenged with 20,000 *E. maxima* oocysts on d 14 and 1 × 10^9^ *C. perfringens* cfu on d 18; T40K^+^, challenged with 40,000 *E. maxima* oocysts on d 14 and 1 × 10^9^ *C. perfringens* cfu on d 18; L, linear effects; Q, quadratic effects. **(A)** Relative frequency of cecal Actinobacteria. **(B)** Relative frequency of cecal Bacteroidetes. **(C)** Relative frequency of cecal Firmicutes. **(D)** Relative frequency of cecal Proteobacteria. **(E)** Relative frequency of cecal Christensenellaceae. **(F)** Relative frequency of cecal Enterobacteriaceae. **(G)** Relative frequency of cecal Lachnospiraceae. **(H)** Relative frequency of cecal Ruminococcaceae.

The taxonomic compositions of cecal bacterial communities at the family and phylum levels are depicted in Figures 9, 10. Comparing the Control group and the T0^+^ group, there were no statistical differences in the relative frequency of major bacterial groups at the family and phylum levels. At the phylum level, the presence of increasing levels of *E. maxima* oocyst dose with *C. perfringens* coinfection led to a significant decrease in the relative frequency of *Actinobacteria* (linear and quadratic, *p* < 0.05) and *Firmicutes* (linear, *p* < 0.05), while *Bacteroidetes* showed an increase (linear, *p* < 0.05) in cecal bacterial communities at 6 dpi. At the family level, the increasing levels of *E. maxima* oocyst dose with *C. perfringens* coinfection resulted in a significant decrease in the relative frequency of *Christensenellaceae* (linear, *p* < 0.05), and a quadratic change was observed in *Lachnospiraceae* (quadratic, *p* < 0.05) in cecal bacterial communities at 6 dpi. No statistical differences were found in the relative frequency of bacteria in all treatment groups.

## 4 Discussion

In this paper, we evaluated the effect of different *E. maxima* doses on the severity of NE to develop a reliable NE model that can be used to evaluate feed additives in commercial broiler chickens. The NE model that we used was a dual infection model using *E. maxima* strain 41A and *C. perfringens* strain Del-1 based on our previous NE studies (Park et al., 2022; Goo et al., 2023a). The *C. perfringens* strain used in this study carries the principal virulence toxin NetB (Goo et al., 2023b), and a group challenged with only *C. perfringens* (T0^+^) without *E. maxima* pre-exposure was also included as a control. To avoid potential interference, no additional anti-coccidial drugs or vaccines were administered, ensuring an accurate assessment of the impact of *E. maxima* levels. Consistent with previous NE model (Goo et al., 2023a), a slightly increased crude protein diet (24%) was provided upon *C. perfringens* inoculation on d 18 (4 dpi from *E. maxima* challenge). In most parameters, there were no significant differences between the Control group and the T0^+^ group in the current study. The parameters that we evaluated included growth performance, intestinal permeability, jejunal NE lesion scores, AID, fecal *E. maxima* oocyst count, jejunal and ileal VH:CD and GC count, gene expression of tight junction proteins (TJPs), host defense peptides (HDPs), and cytokines, alpha-diversity, and the relative frequency of cecal bacterial communities at the phylum and family levels. However, the *C. perfringens* colony count in the intestine showed that the T0^+^ group had approximately 520 times higher jejunal *C. perfringens* counts (log_10_^4.49^ vs. log_10_^7.20^ cfu/mL of digesta) than the Control group. This finding is consistent with previous studies (Du et al., 2016; Fasina and Lillehoj, 2019; Lee et al., 2022a) and indicates the need of other predisposing factors to develop reliable NE model. Although successful colonization of NetB-positive *C. perfringens* was confirmed in the intestine, it did not have direct negative effects such as decreased growth performance or increased mortality, as observed in previous studies (Fasina and Lillehoj, 2019; Lee et al., 2022a). This highlights the fact that positive confirmation of NetB by PCR testing, as shown in the current study and Lee et al. (2011), does not guarantee NE pathogenesis but a reliable NE model requires successful colonization of NetB-positive *C. perfringens* and the presence of a predisposing infection such as *E. maxima* infection.

The measurement of growth performance can be divided into two distinct periods: the acute challenge period from the date of the first *E. maxima* inoculation (d 14) to the expected peak infection effect of NE (d 21, 7 dpi), and the recovery period from 8 to 14 dpi (d 22–28). During the acute challenge period (0–7 dpi), growth performance measurements, including BWG, FI, and FCR, linearly decreased as the levels of *E. maxima* increased. However, no significant differences were observed during the recovery period (8–14 dpi). When considering the entire experimental period (d 14–28), all growth performance parameters decreased as the level of *E. maxima* increased in the current study. According to the results of the DFI trend, the Control group and the T0^+^ group showed no significant difference throughout the entire experimental period and showed a steady increase. The groups challenged with *E. maxima* (T5K^+^, T10K^+^, T20K^+^, and T40K^+^) showed decreased DFI at 5 dpi, with the most substantial decrease observed at 7 dpi. T20K^+^ and T40K^+^ groups showed the most severe reduction in DFI. Subsequently, all the *E. maxima-*challenged groups showed a rapid recovery in DFI. However, they were unable to completely recover the level of decreased FI observed during the acute challenge period (0–7 dpi).

Jejunal NE lesions were assessed on 5, 6, 7, and 14 dpi to verify the NE infection pattern and determine which dpi and *E. maxima* inoculation dose exerted the most severe NE effect in the current study. Except for the last day (14 dpi), all *E. maxima* with *C. perfringens* infection groups showed clear NE lesions compared to the Control group. This finding aligns with the DFI trend observed in this study, where DFI was significantly reduced by coinfection of *E. maxima* with *C. perfringens* from 5 to 7 dpi, but not at 14 dpi. In addition, the NE lesion scores gradually increased from 5 to 7 dpi in most groups, and NE lesions showed a linear increase as the level of *E. maxima* increased on 5, 6, and 7 dpi. The reproductive cycle of *E. maxima* is likely the main reason behind these significant trends in both DFI and NE lesion scores. Following *Eimeria* infection, maximum intestinal damage occurs around 4–6 dpi during the gametogony stage (Peek, 2010). Sporozoites, upon invasion of intestinal cells, transform into schizonts through asexual reproduction, and merozoites are released from the schizonts around 5 dpi, causing substantial damage to the intestine (Teng et al., 2020). Merozoites continue to reproduce asexually, damaging intestinal cells for their reproductive activity (McDougald, 1998) which is demonstrated by the occurrence of severe NE lesions in the intestine starting from 5 dpi (Gholamiandehkordi et al., 2007). As a result of repeated destruction of the intestinal barrier and increased protein leakage, *C. perfringens* finds optimal conditions for colonization (Van Immerseel et al., 2004), leading to maximal NE lesions around 7 dpi. This result indirectly explains why most recent NE experiments selected 7 dpi for lesion measurement (Akerele et al., 2022; Park et al., 2022; Pham et al., 2022). Additionally, in the current study, decreased growth performance was observed in broilers with NE lesions but without mortality (no mortality after d 14 in all groups), which is considered a successful subclinical NE model.

The results of serum intestinal permeability also showed a linear increase as the infecting dose of *E. maxima* level increased at 5, 6, and 7 dpi. This finding was similar to the NE lesion scores result although the highest intestinal permeability was observed at 6 dpi. A previous NE study that used *E. maxima* as a predisposing factor, also reported the highest intestinal permeability at 6 dpi (Akerele et al., 2022). Nevertheless, the decrease in intestinal permeability between 6 and 7 dpi was quite significant in the current study, even though fecal *E. maxima* oocyst counts peaked at 7 dpi and a significant number of *E. maxima* were still found in the villi at 6 dpi. This dramatic change in intestinal permeability is reflected by our intestinal permeability analysis using FITC-d, a more direct measurement of gut damage and intestinal integrity compared to NE lesion scoring or fecal oocyst counting. This pattern of change in intestinal permeability is also believed to be closely related to the reproduction cycle of *Eimeria*, as mentioned in previous studies (Teng et al., 2020; Sharma et al., 2022). This emphasizes the important role of *Eimeria* as a predisposing factor in our NE model, and the current study confirms the importance of *E. maxima* pre-exposure for reliable NE model as observed in previous studies (Park et al., 2008).

Fecal *E. maxima* oocysts showed a linear increase with increasing levels of infecting *E. maxima* oocyst at 6, 7, and 14 dpi, but no oocysts were found at 5 dpi. Additionally, on 7 dpi, jejunal *C. perfringens* colony counts linearly increased with the level of *E. maxima* in the NE condition. Under conditions that do not exceed a saturation threshold, it is well-documented that fecal *E. maxima* levels increase depending on the level of *E. maxima* inoculation (Teng et al., 2021). Previous studies have reported a numerical increase in *C. perfringens* in the intestine of the *Eimeria* and *C. perfringens* co-infection groups compared to the *C. perfringens* challenge alone group (Park et al., 2008; Wu et al., 2010). In the current study, the T40K^+^ group exhibited approximately 10 times more jejunal *C. perfringens* counts than the T0^+^ group (log_10_^7.20^ vs. log_10_^8.25^ cfu/mL of digesta). This is because *E. maxima* causes gut inflammation, serum leakage, and mucogenesis, creating optimal conditions for *C. perfringens* colonization (Forder et al., 2012; Adhikari et al., 2020). Therefore, as the level of *E. maxima* increased, *C. perfringens* effectively colonized the intestines under the more favorable conditions caused by *E. maxima*. Thus, the increased level of *E. maxima* effectively facilitated the colonization of *C. perfringens* in the present study, further confirming the crucial role of *E. maxima* as a predisposing factor in establishing the NE model as long as the infection doses of *E. maxima* are not overtly destructive.

In the current subclinical NE condition, the AID of DM, OM, ash, and crude protein all showed a linear decrease, while ileal and fecal water content linearly increased as the *E. maxima* level increased. Previous studies have reported that NE causes destruction of the intestinal mucosa, leading to decreased digestibility of crude protein (Zanu et al., 2020; Kumar et al., 2021a). Moreover, impaired intestinal mucosal function due to direct intestinal damage hampers nutrient utilization, particularly amino acid absorption and metabolism, with increased *Eimeria* infection levels (Paiva et al., 2013; Rochell et al., 2016). Undigested nutrients in the intestine can also provide a substrate for the proliferation of pathogenic bacteria, notably aiding the colonization of *C. perfringens* (Paiva et al., 2013). Therefore, in the current study, the reduced nutrient absorption ability due to increased *E. maxima* levels may have contributed to both decreased growth performance and increased *C. perfringens* colonization in chickens. NE causes direct damage to the intestine, resulting in distinct NE lesions and symptoms such as diarrhea (Songer and Meer, 1996). The current study observed an increase in water content as the *E. maxima* level increased, consistent with a previous NE-related study reporting an increase in diarrhea score with higher *Eimeria* spp. levels (Lee et al., 2022b). When diarrhea occurs, nutrients in the large intestine cannot be properly absorbed and are excreted from the body (Bilgili et al., 2010). Given the intestinal damage and reduced AID observed in the NE infection in this study, it is highly probable that the higher water content in the chicken’s intestine did not facilitate optimal absorption. However, it is important to note that although diarrhea is typically recognized as a clinical sign of NE (Shojadoost et al., 2012), insufficient evidence exists to classify the increased moisture content in the intestine as diarrhea in this study, as it was identified as a clear subclinical NE.

Intestinal morphology provides a more direct means of identifying changes in the intestine resulting from NE infection. In this study, as the *E. maxima* level increased, both jejunum and ileum showed a linear decrease in VH:CD and GC count per villus. This finding complements the result that NE infection had negative effects on the chickens. Reduced VH:CD and GC numbers indicate a deterioration in overall intestinal health, resulting in decreased nutrient absorption capacity and compromised mucosal barrier integrity (McGuckin et al., 2011; Mora et al., 2020). Similar to previous studies (Lee et al., 2022b; Pham et al., 2022), the continuous turnover of crypt cells in response to inflammation caused by pathogens such as *E. maxima* and *C. perfringens* leads to increased crypt depth (Paiva et al., 2014). Furthermore, various pathogens attempt to penetrate the mucin layer, and a decrease in the number of GCs can facilitate their infiltration into epithelial cells (McGuckin et al., 2011). Therefore, NE triggers local immune responses in the early stages that consume energy for epithelial cell recovery, ultimately leading to an overall decrease in growth (Laudadio et al., 2012).

The gene expression results in the current experiment encompass several major categories: TJPs, nutrient transporters, HDPs, pro- and anti-inflammatory cytokines, and *E. maxima* gametocytes. Intestinal epithelial cells play a crucial role in protecting the host from various pathogens, with TJPs acting as proteins that maintain the integrity of the gaps between these cells (Awad et al., 2017). In addition, the mucin secreted by GC forms a protective mucus layer on the epithelial cells, primarily defending the host against pathogenic bacteria (McGuckin et al., 2011). In this study, CLDN1 showed a linear upregulation as the *E. maxima* level increased, while MUC2 showed a linear downregulation. The CLDN family comprises integral membrane proteins that regulate paracellular transport in epithelial cells (Awad et al., 2017). Despite their vital role in tight junction complexes, the expression of CLDN1 increased, similar to findings from previous studies (Bortoluzzi et al., 2019; Gharib-Naseri et al., 2021). Although TJP genes are typically downregulated during infection, one study suggests that the upregulation of CLDN1 may not universally indicate increased barrier function (Bhat et al., 2020). Previous studies have reported that TNF-α increases the expression of CLDN1 in human epithelial cells (Poritz et al., 2011), and the possibility of increased expression following infection was also mentioned due to the anti-apoptotic effect of CLDN1 (Akasaka et al., 2010; Gharib-Naseri et al., 2021). However, the specific mechanism by which CLDN1 expression increases during NE infection in chickens is not yet fully understood. The downregulation of MUC2 can be attributed to a decrease in the number of GCs. The downregulation of MUC2 increases bacterial translocation and inflammation, and NE infection can damage the intestinal epithelial layer, making it more susceptible to further pathogenic infections (Wei et al., 2012). Similarly to the CLDN family, OCLN and the ZO family are involved in regulating paracellular and intermembrane diffusion (Ulluwishewa et al., 2011). However, the expression of OCLN and ZO2 in the current study showed quadratic regulation as the *E. maxima* level increased. This pattern is believed to be influenced by the level of *Eimeria*. Similar findings have been reported in previous studies where the infecting dose of *Eimeria* oocysts were intentionally increased (Teng et al., 2020; 2021). One possible explanation for this phenomenon is that when the *E. maxima* level surpasses a certain threshold, there may be limited space for the continuation for the reproduction cycle (Williams, 2001).

In the current study, the majority of nutrient transporters (B^0^AT, B^0,+^AT, PepT1, and GLUT2) showed a linear downregulation as the level of *E. maxima* increased, while the expression of EAAT3 showed a quadratic change. These results reflect the complex negative effects of NE infection. Nutrient transporters located on the plasma membrane of the small intestine play a crucial role in regulating amino acid and energy intake, which are essential for animal immune function and disease resistance (Gharib-Naseri et al., 2021). B^0^AT and B^0,+^AT are responsible for transporting a wide range of neutral and cationic amino acids, respectively (Speier et al., 2012; Fotiadis et al., 2013). PepT1, a peptide transporter, facilitates the transport of luminal amino acids in the form of di- and tripeptides (Smith et al., 2013). GLUT2, a monosaccharide carrier, transports glucose, galactose, fructose, and other sugars from outside the cell into the bloodstream (Mueckler and Thorens, 2013). Therefore, the downregulation of these nutrient transporters directly results in decreased nutrient absorption necessary for animal immunity against pathogens or diseases, ultimately impacting productivity as the expression of nutrient transporters decreases. However, it remains unclear why the T0^+^ group (*C. perfringens* challenged only) showed an upregulation of GLUT2 compared to the Control group in the current study. The expression of EAAT3, which transports glutamate, the main energy source for intestinal epithelial cells (Su et al., 2014), exhibited a quadratic change. As mentioned earlier, this may be attributed to the crowding effect of *E. maxima* (Williams, 2001; Teng et al., 2021), particularly concerning the gene expression patterns of TJPs and nutrient transporters that show quadratic responses (OCLN, ZO2, MUC2, B^0,+^AT, EAAT3, and GLUT2). Interestingly, the highest or lowest values were observed at T20K^+^ rather than T40K^+^. However, considering the results of growth performance and intestinal permeability, it is challenging to conclude that the effect of NE decreased in the T40K^+^ group compared to the T20K^+^ group. This discrepancy could be due to sampling deviation caused by a decrease in FI resulting from a severe NE infection in the T40K^+^ group. Therefore, a broader range of *E. maxima* levels is required to determine the exact crowding effect in NE experiments.

In avian species, there are three primary classes of HDPs: AvBDs, CATHLs, and LEAP2 (Lynn et al., 2003; Van Dijk et al., 2008; 2011). Beta-defensins are small cationic peptides that play a crucial role in the innate immune system and are responsible for regulating bacterial growth (Cooper et al., 2019). AvBD3, expressed in the gastrointestinal tract, bursa, thymus, and spleen, has been shown to inhibit the growth of gram-positive bacteria (Ma et al., 2013; Cooper et al., 2019). AvBD9, an important HDP, is widely present in the gastrointestinal mucosa and skin of chickens and plays a vital role in host immunity and defense against infections (Liu et al., 2019). In the current study, both AvBD3 and AvBD9 showed a linear downregulation as *E. maxima* level increased under NE conditions. Previous study reported a downregulation of AvBD3 in the intestinal mucosal layer of NE-infected chickens (Hong et al., 2012), and Yang et al. (2022) observed a downregulation of AvBD9 in the chicken jejunum during *C. perfringens* infection. Certain bacteria are known to weaken the host’s immune response and downregulate the expression of beta-defensins to facilitate successful infection (Sperandio et al., 2008; Meade et al., 2009). Hence, the downregulation of AvBD3 and AvBD9 observed in this study may be a result of *C. perfringens*, which coexisted with increased levels of *E. maxima*, evading the host’s innate immunity mediated by HDPs to establish infection. Chicken LEAP2 is highly expressed in epithelial tissues and primarily functions to protect from the invasion or adhesion of pathogenic microbes (Townes et al., 2004). LEAP2 expression is known to be downregulated during *E. maxima* infection (Casterlow et al., 2011), which aligns with the findings of the current study. Interestingly, LEAP2 is upregulated in response to bacterial infections such as *Salmonella* (Townes et al., 2004). In the current experiment, the expression of LEAP2 in the group challenged with *C. perfringens* alone was higher than that in the other infection groups with both *C. perfringens* and *E. maxima*. This result suggests that the regulation of LEAP2 expression may differ depending on the type of pathogen infection. Parasites like *E. maxima* appear to downregulate LEAP2 expression, while bacterial pathogens like *C. perfringens* upregulate it. Therefore, the downregulation of LEAP2 during *E. maxima* infection makes the host’s intestine more vulnerable to additional pathogen infections such as *C. perfringens* (Tian et al., 2016). It is well-established that CATHLs are highly expressed in immune organs and the intestine (Cheng et al., 2015), and their expression is induced by microbial infections such as *Salmonella* and *C. perfringens*, as well as inflammatory cytokines (Akbari et al., 2008; Tian et al., 2016). Unlike other HDPs such as AvBDs and LEAP2, the gene expression of CATHL3 showed a linear upregulation with increasing *E. maxima* levels. Tian et al. (2016) reported an increased in the expression of CATHL1 and 2 during NE infection, which could contribute to effective resistance against *E. maxima* and *C. perfringens*. Because the four avian CATHL types (CATHL1, 2, 3, and CATHB1) share similar structures (Cheng et al., 2015), the upregulation of CATHL3 observed in this study might play a similar protective role in the host, akin to CATHL1 and 2. However, the exact reasons for the distinct expression patterns compared to other HDPs (AvBDs and LEAP2) remain unclear.

The inflammatory response is regulated by complex endogenous cell signaling pathways (Takeuchi and Akira, 2010). Upon recognition of pathogen-associated molecular patterns (PAMPs) by pathogen recognition receptors (PRRs) such as TLRs, the host’s innate immune response is initiated (Palm and Medzhitov, 2009). Activation of the NF-κB signaling pathway occurs via TLRs upon PAMP recognition, leading to the production of inflammatory cytokines such as IL-1β, IL-6, and IFN-γ (Akira and Takeda, 2004). In the present study, the expression of TLR2 and NF-κB was not affected by NE, whereas TLR4 showed a linear downregulation with increasing *E. maxima* levels. Wu et al. (2018) suggested that the difference in TLR4 and cytokine mRNA expression could be attributed to variations in sampling time and pathogen load in the intestines of chickens infected with NE. Furthermore, a study on *E. praecox* infection reported downregulation of TLR4 expression (Sumners et al., 2011). Hence, our results indicate that the observed downregulation of TLR4 is likely influenced by the *E. maxima* levels rather than *C. perfringens*.

Cytokines play a crucial role in the animal immune system (Lin and Karin, 2007; Lu et al., 2022). Both *Eimeria* and *C. perfringens* activates both innate and adaptive immune responses in chickens, leading to the secretion of various cytokines (Fasina and Lillehoj, 2019; Lee et al., 2022c). IL-1β, a potent pro-inflammatory cytokine mainly secreted by activated macrophages, plays a vital role in innate responses to infections (Lopez-Castejon and Brough, 2011). IFN-γ, a key mediator of the innate and adaptive immune system, is produced by T cells and natural killer (NK) cells and is involved in functions such as macrophage activation and antibacterial immunity (Tau and Rothman, 1999). TNF-α, another pleiotropic pro-inflammatory cytokine, mediates cell survival and pro-inflammatory responses through the NF-κB pathway upon binding to TNF receptor 1 (Bradley, 2008). TNFSF15, also known as TNF ligand-related molecule 1, contributes to the innate and adaptive immune response in mucosal tissues against bacterial infections by binding to its specific receptor (Kadiyska et al., 2018). On the other hand, IL-10, an anti-inflammatory cytokine, is produced by various leukocytes, including T cells, macrophages, neutrophils, dendritic cells, and NK cells, and shares functional similarities with IFN-γ (Savan et al., 2009; Kany et al., 2019). Contrary to the results for TLR4, most pro-inflammatory cytokines (IL-1β, IFN-γ, TNFSF15, and TNF-α) and the anti-inflammatory cytokine IL-10 were found to be linearly upregulated with increasing *E. maxima* levels in the current study. These findings suggest that NE induces a wide-spectrum local inflammatory response, particularly when *E. maxima* is a predisposing factor. However, as mentioned earlier, the typical process of inducing inflammatory cytokines to protect the host against pathogens occurs through the NF-κB signaling pathway triggered by TLRs (Akira and Takeda, 2004). It is noteworthy that TLR4 and inflammatory cytokines exhibited opposite expression patterns in the current study. One possible explanation for this discrepancy is that the upregulation of TLR4 may not be necessary to initiate inflammatory responses in the intestine due to the tight regulation of TLR4 expression and activity by a regulatory system called Toll-interacting protein (Hubbard and Moore, 2010; Shojadoost et al., 2022). Further research is needed to understand how NE, generated by *E. maxima* and *C. perfringens* coinfection, affects the relationship between TLRs and inflammatory cytokines, especially considering that the expression of NF-κB did not show any significant difference in the current study. In chickens, IL-2 generally plays a role in T cell proliferation, NK cell activation, and elimination of intracellular pathogens (Kaiser and Mariani, 1999; Susta et al., 2015). However, in the current study, only IL-2 was downregulated in all challenged groups compared to the Control group. A previous study reported that IL-10 was upregulated when IL-2 was downregulated in NE-challenged conditions (Wang et al., 2017). This may be because IL-10, with its immunoregulatory role in the intestine, inhibits the synthesis of pro-inflammatory cytokines, thereby downregulating inflammatory Th1 responses (Groux and Powrie, 1999; Rothwell et al., 2004).

*E. maxima* gametocyte-related genes (APN, EF2, Gam56, Gam82, and IMC1) were utilized to assess *Eimeria* reproduction activities in the intestine, and differences among the four coinfection groups (T5K^+^, T10K^+^, T20K^+^, and T40K^+^) were observed in the present experiment. As the level of *E. maxima* increased, two genes associated with macrogametocyte wall formation (Gam56 and Gam82) exhibited a linear upregulation (Walker et al., 2015). Furthermore, the expression of Gam82 was highest in the T20K^+^ group, and Gam56 also demonstrated the highest numerical value among the four *E. maxima* challenged groups reflecting a significant occurrence of oocyst wall formation and *E. maxima* macrogametocyte formation (Krucken et al., 2008). The highest expression of Gam82 in the T20K^+^ group may suggest the presence of a potential threshold due to the crowding effect in T20K^+^ and T40K^+^ groups.

NE induces changes in the microbiota of the small intestine through modulation of the host’s immune response (Antonissen et al., 2016). Moreover, high levels of *Eimeria* can significantly impact the reduction of intestinal microbiota diversity by decreasing the abundance of operational taxonomic units (OTUs) and increasing the dominance of the bacterial community (Perez et al., 2011). The cecal bacterial composition on 6 dpi indicated that the alpha-diversity indices, including Pielou’s evenness and Shannon’s entropy, are similar and as the level of *E. maxima* increased with *C. perfringens*, the diversities decreased linearly, with the T40K^+^ group demonstrating the lowest diversities. The alpha-diversity results in the current study indicate that our dual-infection model of NE disturbed normal gut microflora whereas no significant alteration of alpha-diversity indices between the Control group and the group challenged with *C. perfringens* alone. This result is consistent with previous studies that suggested the role of a predisposing infection in NE (Stanley et al., 2014; Feng et al., 2022). Significant differences were also observed in the beta-diversity indices (unweighted UniFrac and Jaccard distance) between the Control group and all groups challenged with *C. perfringens*. This suggests that the inoculation of *C. perfringens* caused substantial changes in cecal microbiota compared to the Control group. These variations in diversities may be attributed to unstable changes in the intestinal microbiota caused by the presence of pathogens.

The intestinal microbial community is a highly complex ecosystem that interacts with the host’s health through nutritional, physiological, and immunological factors (Kumar et al., 2021b). In normal chickens, the cecal microbiota is predominantly composed of *Firmicutes*, *Bacteroidetes*, and *Proteobacteria* (Oakley et al., 2014; Lin et al., 2017), which was consistent across all groups. In the present study, however, the abundance of *Actinobacteria* and *Firmicutes* showed a linear decrease at the phylum level, while Bacteroidetes showed a linear increase with higher levels of *E. maxima* and *C. perfringens*. The reduction in the relative abundance of *Firmicutes* and the decrease in the *Firmicutes*-to-*Bacteroidetes* ratio (from 3.9 to 1.5 for T0^+^ and T40K^+^, respectively) due to NE infection can negatively impact energy utilization and disrupt proper physiological and nutritional balance (Zhang et al., 2018). At the family level, the abundance of *Christensenellaceae* and *Lachnospiraceae* was influenced by the increased presence of *E. maxima* and *C. perfringens*. *Christensenellaceae* has been reported to play a crucial role in intestinal health (Mancabelli et al., 2017), while *Lachnospiraceae* is a major producer of butyrate, which is an anti-inflammatory metabolite that helps maintain intestinal integrity (Eeckhaut et al., 2011). The production of butyrate by *Lachnospiraceae* potentially reduces the incidence and severity of NE, as this family may primarily colonize the ceca in broilers and inhibit *C. perfringens* growth (Antonissen et al., 2016). Therefore, the changes observed in *Lachnospiraceae* abundance in the current study may have been influenced by the activity of administered *C. perfringens* and the presence of NE infection.

In summary, increasing levels of *E. maxima* are associated with heightened NE gut lesion, fecal *E. maxima* oocyst counts, and intestinal *C. perfringens* colonization. These changes led to increased intestinal permeability, altered expression of TJPs genes, downregulation of nutrient transporters and digestibility, decreased GC numbers and VH:CD, and downregulation of MUC2, which collectively hindered nutrient absorption in the intestine and compromised host defense system. Furthermore, downregulation of HDPs, upregulation of pro- and anti-inflammatory cytokines, decreased intestinal microbial diversity, and microbial imbalance in NE-affected broilers resulted in increased energy consumption for immune response and increased susceptibility to NE, ultimately leading to decreased overall growth performance. Notably, the challenge with *C. perfringens* alone, without *E. maxima*, did not induce NE symptoms in this study, further emphasizing the importance of *E. maxima* as a predisposing factor for NE. Some parameters exhibited a quadratic relationship with the level of *E. maxima*, and the T20K^+^ group showed the maximum effects, suggesting the existence of an optimal range of *E. maxima* inoculation dosage as a predisposing factor for NE.

In conclusion, this study documents the importance of *Eimeria* as a predisposing factor for NE and the influence of infection doses on the severity of disease outcome. These findings underscore the importance of predisposing factors in the pathogenesis of NE.

## Data Availability

The datasets presented in this study can be found in online repositories. The names of the repository/repositories and accession number(s) can be found in the article/Supplementary Material.

## References

[B1] Adhikari P. Kiess A. Adhikari R. Jha R. (2020). An approach to alternative strategies to control avian coccidiosis and necrotic enteritis. J. Appl. Poult. Res. 29, 515–534. 10.1016/j.japr.2019.11.005

[B2] Akasaka H. Sato F. Morohashi S. Wu Y. Liu Y. Kondo J. (2010). Anti-apoptotic effect of claudin-1 in tamoxifen-treated human breast cancer MCF-7 cells. BMC cancer 10, 1–13. 10.1186/1471-2407-10-548 20937153 PMC2958956

[B3] Akbari M. R. Haghighi H. R. Chambers J. R. Brisbin J. Read L. R. Sharif S. (2008). Expression of antimicrobial peptides in cecal tonsils of chickens treated with probiotics and infected with *Salmonella enterica* serovar typhimurium. Clin. Vaccine Immunol. 15, 1689–1693. 10.1128/CVI.00242-08 18827189 PMC2583514

[B4] Akerele G. Al Hakeem W. G. Lourenco J. Selvaraj R. K. (2022). The effect of necrotic enteritis challenge on production performance, cecal microbiome, and cecal tonsil transcriptome in broilers. Pathogens 11, 839. 10.3390/pathogens11080839 36014961 PMC9414309

[B5] Akira S. Takeda K. (2004). Toll-like receptor signalling. Nat. Rev. Immunol. 4, 499–511. 10.1038/nri1391 15229469

[B6] Annett C. B. Viste J. R. Chirino-Trejo M. Classen H. L. Middleton D. M. Simko E. (2002). Necrotic enteritis: effect of barley, wheat and corn diets on proliferation of *Clostridium perfringens* type A. Avian Pathol. 31, 598–601. 10.1080/0307945021000024544 12593744

[B7] Antonissen G. Eeckhaut V. Van Driessche K. Onrust L. Haesebrouck F. Ducatelle R. (2016). Microbial shifts associated with necrotic enteritis. Avian Pathol. 45, 308–312. 10.1080/03079457.2016.1152625 26950294

[B8] Arora S. Gordon J. Hook M. (2021). Collagen binding proteins of gram-positive pathogens. Front. Microbiol. 12, 628798. 10.3389/fmicb.2021.628798 33613497 PMC7893114

[B125] Awad W. A. Hess C. Hess M. (2017). Enteric pathogens and their toxin-induced disruption of the intestinal barrier through alteration of tight junctions in chickens. Toxins 9, 60. 10.3390/toxins9020060 28208612 PMC5331439

[B9] Bhat A. A. Syed N. Therachiyil L. Nisar S. Hashem S. Macha M. A. (2020). Claudin-1, a double-edged sword in cancer. Int. J. Mol. Sci. 21, 569. 10.3390/ijms21020569 31952355 PMC7013445

[B124] Bilgili S. F. Hess J.B. Donald J. Fancher B. (2010). Practical considerations for reducing the risk of pododermatitis. Aviagen Brief 1, 1–8.

[B10] Bolyen E. Rideout J. R. Dillon M. R. Bokulich N. A. Abnet C. C. Al-Ghalith G. A. (2019). Reproducible, interactive, scalable and extensible microbiome data science using QIIME 2. Nat. Biotechnol. 37, 852–857. 10.1038/s41587-019-0209-9 31341288 PMC7015180

[B11] Bortoluzzi C. Vieira B. S. Lumpkins B. Mathis G. F. King W. D. Graugnard D. (2019). Can dietary zinc diminish the impact of necrotic enteritis on growth performance of broiler chickens by modulating the intestinal immune-system and microbiota? Poult. Sci. 98, 3181–3193. 10.3382/ps/pez045 31220319

[B12] Bradley J. (2008). TNF‐mediated inflammatory disease. J. Pathology A J. Pathological Soc. G. B. Irel. 214, 149–160. 10.1002/path.2287 18161752

[B13] Calefi A. S. Honda B. T. B. Costola-de-Souza C. de Siqueira A. Namazu L. B. Quinteiro-Filho W. M. (2014). Effects of long-term heat stress in an experimental model of avian necrotic enteritis. Poult. Sci. 93, 1344–1353. 10.3382/ps.2013-03829 24879684

[B14] Casterlow S. Li H. Gilbert E. R. Dalloul R. A. McElroy A. P. Emmerson D. A. (2011). An antimicrobial peptide is downregulated in the small intestine of Eimeria maxima-infected chickens. Poult. Sci. 90, 1212–1219. 10.3382/ps.2010-01110 21597061

[B15] Cheng Y. Prickett M. D. Gutowska W. Kuo R. Belov K. Burt D. W. (2015). Evolution of the avian β-defensin and cathelicidin genes. BMC Evol. Biol. 15, 1–17. 10.1186/s12862-015-0465-3 26373713 PMC4571063

[B16] Choi J. Yadav S. Wang J. Lorentz B. J. Lourenco J. M. Callaway T. R. (2022a). Effects of supplemental tannic acid on growth performance, gut health, microbiota, and fat accumulation and optimal dosages of tannic acid in broilers. Front. Physiology 13, 912797. 10.3389/fphys.2022.912797 PMC9478478 36117708

[B17] Choi J. Tompkins Y. H. Teng P. Y. Gogal R. M. Jr Kim W. K. (2022b). Effects of tannic acid supplementation on growth performance, oocyst shedding, and gut health of in broilers infected with eimeria maxima. Animals 12, 1378. 10.3390/ani12111378 35681844 PMC9179276

[B18] Collier C. T. Hofacre C. L. Payne A. M. Anderson D. B. Kaiser P. Mackie R. I. (2008). Coccidia-induced mucogenesis promotes the onset of necrotic enteritis by supporting *Clostridium perfringens* growth. Veterinary Immunol. Immunopathol. 122, 104–115. 10.1016/j.vetimm.2007.10.014 18068809

[B19] Cooper K. K. Songer J. G. (2009). Necrotic enteritis in chickens: A paradigm of enteric infection by *Clostridium perfringens* type A. Anaerobe 15, 55–60. 10.1016/j.anaerobe.2009.01.006 19186215

[B20] Cooper K. K. Songer J. G. (2010). Virulence of *Clostridium perfringens* in an experimental model of poultry necrotic enteritis. Veterinary Microbiol. 142, 323–328. 10.1016/j.vetmic.2009.09.065 19931323

[B21] Cooper C. A. Tizard M. L. Stanborough T. Moore S. C. Chandry P. S. Jenkins K. A. (2019). Overexpressing ovotransferrin and avian β-defensin-3 improves antimicrobial capacity of chickens and poultry products. Transgenic Res. 28, 51–76. 10.1007/s11248-018-0101-2 30374651

[B22] Du E. Wang W. Gan L. Li Z. Guo S. Guo Y. (2016). Effects of thymol and carvacrol supplementation on intestinal integrity and immune responses of broiler chickens challenged with *Clostridium perfringens* . J. animal Sci. Biotechnol. 7, 19–10. 10.1186/s40104-016-0079-7 PMC4802587 27006768

[B23] Eeckhaut V. Van Immerseel F. Croubels S. De Baere S. Haesebrouck F. Ducatelle R. (2011). Butyrate production in phylogenetically diverse Firmicutes isolated from the chicken caecum. Microb. Biotechnol. 4, 503–512. 10.1111/j.1751-7915.2010.00244.x 21375722 PMC3815262

[B24] Fasina Y. O. Lillehoj H. S. (2019). Characterization of intestinal immune response to *Clostridium perfringens* infection in broiler chickens. Poult. Sci. 98, 188–198. 10.3382/ps/pey390 30239962 PMC6347130

[B25] Feng X. Li T. Zhu H. Liu L. Bi S. Chen X. (2022). Effects of challenge with *Clostridium perfringens*, Eimeria and both on ileal microbiota of yellow feather broilers. Front. Microbiol. 13, 1063578. 10.3389/fmicb.2022.1063578 36532499 PMC9754095

[B26] Forder R. E. A. Nattrass G. S. Geier M. S. Hughes R. J. Hynd P. I. (2012). Quantitative analyses of genes associated with mucin synthesis of broiler chickens with induced necrotic enteritis. Poult. Sci. 91, 1335–1341. 10.3382/ps.2011-02062 22582290

[B27] Fotiadis D. Kanai Y. Palacín M. (2013). The SLC3 and SLC7 families of amino acid transporters. Mol. aspects Med. 34, 139–158. 10.1016/j.mam.2012.10.007 23506863

[B28] Gharib-Naseri K. Kheravii S. Keerqin C. Swick R. A. Choct M. Wu S. B. (2021). Differential expression of intestinal genes in necrotic enteritis challenged broiler chickens with 2 different *Clostridium perfringens* strains. Poult. Sci. 100, 100886. 10.1016/j.psj.2020.11.063 33516477 PMC7936145

[B29] Gholamiandehkordi A. R. Timbermont L. Lanckriet A. Broeck W. V. D. Pedersen K. Dewulf J. (2007). Quantification of gut lesions in a subclinical necrotic enteritis model. Avian Pathol. 36, 375–382. 10.1080/03079450701589118 17899461

[B30] Goo D. Gadde U. D. Kim W. K. Gay C. G. Porta E. W. Jones S. W. (2023a). Hyperimmune egg yolk antibodies developed against *Clostridium perfringens* antigens protect against necrotic enteritis. Poult. Sci. 102, 102841. 10.1016/j.psj.2023.102841 37480657 PMC10393821

[B31] Goo D. Park I. Nam H. Lee Y. Sawall J. Smith A. H. (2023b). Collagen adhesin protein and necrotic enteritis B-like toxin as biomarkers for early diagnosis of necrotic enteritis in commercial broiler chickens. Poult. Sci. 102, 102647. 10.1016/j.psj.2023.102647 37060834 PMC10139936

[B32] Groux H. Powrie F. (1999). Regulatory T cells and inflammatory bowel disease. Immunol. today 20, 442–445. 10.1016/s0167-5699(99)01510-8 10500290

[B33] Hermans P. G. Morgan K. L. (2007). Prevalence and associated risk factors of necrotic enteritis on broiler farms in the United Kingdom; a cross-sectional survey. Avian Pathol. 36, 43–51. 10.1080/03079450601109991 17364509

[B34] Hong Y. H. Song W. Lee S. H. Lillehoj H. S. (2012). Differential gene expression profiles of β-defensins in the crop, intestine, and spleen using a necrotic enteritis model in 2 commercial broiler chicken lines. Poult. Sci. 91, 1081–1088. 10.3382/ps.2011-01948 22499864

[B35] Hubbard L. L. Moore B. B. (2010). IRAK-M regulation and function in host defense and immune homeostasis. Infect. Dis. Rep. 2, e9. 10.4081/idr.2010.e9 21390243 PMC3049547

[B36] Kadiyska T. Tourtourikov I. Popmihaylova A. M. Kadian H. Chavoushian A. (2018). Role of TNFSF15 in the intestinal inflammatory response. World J. Gastrointest. Pathophysiol. 9, 73–78. 10.4291/wjgp.v9.i4.73 30809418 PMC6384511

[B37] Kaiser P. Mariani P. (1999). Promoter sequence, exon: intron structure, and synteny of genetic location show that a chicken cytokine with T-cell proliferative activity is IL2 and not IL15. Immunogenetics 49, 26–35. 10.1007/s002510050460 9811966

[B38] Kany S. Vollrath J. T. Relja B. (2019). Cytokines in inflammatory disease. Int. J. Mol. Sci. 20, 6008. 10.3390/ijms20236008 31795299 PMC6929211

[B39] Krucken J. Hosse R. J. Mouafo A. N. Entzeroth R. Bierbaum S. Marinovski P. (2008). Excystation of Eimeria tenella sporozoites impaired by antibody recognizing gametocyte/oocyst antigens GAM22 and GAM56. Eukaryot. Cell. 7, 202–211. 10.1128/EC.00292-07 18083827 PMC2238154

[B40] Kumar A. Toghyani M. Kheravii S. K. Pineda L. Han Y. Swick R. A. (2021a). Potential of blended organic acids to improve performance and health of broilers infected with necrotic enteritis. Anim. Nutr. 7, 440–449. 10.1016/j.aninu.2020.11.006 34258432 PMC8245907

[B41] Kumar A. Kheravii S. K. Ionescu C. Blanchard A. Barekatain R. Bajagai Y. S. (2021b). A microencapsulated mixture of eugenol and garlic tincture supplementation mitigates the effect of necrotic enteritis on intestinal integrity and increases goblet cells in broilers. Microorganisms 9, 1451. 10.3390/microorganisms9071451 34361887 PMC8303895

[B42] Laudadio V. Passantino L. Perillo A. Lopresti G. Passantino A. Khan R. U. (2012). Productive performance and histological features of intestinal mucosa of broiler chickens fed different dietary protein levels. Poult. Sci. 91, 265–270. 10.3382/ps.2011-01675 22184453

[B43] Lee K. W. Lillehoj H. S. (2021). Role of *Clostridium perfringens* necrotic enteritis B-like toxin in disease pathogenesis. Vaccines 10, 61. 10.3390/vaccines10010061 35062722 PMC8780507

[B44] Lee K. W. Lillehoj H. S. Jeong W. Jeoung H. Y. An D. J. (2011). Avian necrotic enteritis: experimental models, host immunity, pathogenesis, risk factors, and vaccine development. Poult. Sci. 90, 1381–1390. 10.3382/ps.2010-01319 21673152

[B45] Lee K. W. Lillehoj H. S. Kim W. Park I. Li C. Lu M. (2021). Research note: first report on the detection of necrotic enteritis (NE) B-like toxin in biological samples from NE-afflicted chickens using capture enzyme-linked immunosorbent assay. Poult. Sci. 100, 101190. 10.1016/j.psj.2021.101190 34087701 PMC8182422

[B46] Lee H. G. Kim Y. B. Lee S. H. Moon J. O. Chae J. P. Kim Y. J. (2022a). *In vivo* recovery of bacteriophages and their effects on Clostridium perfringens-infected broiler chickens. Veterinary Sci. 9, 119. 10.3390/vetsci9030119 PMC8953289 35324847

[B47] Lee J. H. Lee B. Rousseau X. Gomes G. A. Oh H. J. Kim Y. J. (2022b). Stimbiotic supplementation modulated intestinal inflammatory response and improved broilers performance in an experimentally-induced necrotic enteritis infection model. J. Animal Sci. Biotechnol. 13, 100–117. 10.1186/s40104-022-00753-9 PMC9472449 36100948

[B48] Lee Y. Lu M. Lillehoj H. S. (2022c). Coccidiosis: recent progress in host immunity and alternatives to antibiotic strategies. Vaccines 10, 215. 10.3390/vaccines10020215 35214673 PMC8879868

[B49] Lee Y. Park I. Lillehoj H. S. (2023). Oral administration of chicken NK-lysin or recombinant chicken IL-7 improves vaccine efficacy of Eimeria tenella elongation factor-1α (EF-1α) against coccidiosis in commercial broiler chickens. Poult. Sci. 102, 102611. 10.1016/j.psj.2023.102611 36940651 PMC10036930

[B50] Lepp D. Zhou Y. Ojha S. Mehdizadeh Gohari I. Carere J. Yang C. (2021). *Clostridium perfringens* produces an adhesive pilus required for the pathogenesis of necrotic enteritis in poultry. J. Bacteriol. 203, e00578-20–e01128. 10.1128/JB.00578-20 33468589 PMC8088525

[B51] Lin W. W. Karin M. (2007). A cytokine-mediated link between innate immunity, inflammation, and cancer. J. Clin. investigation 117, 1175–1183. 10.1172/JCI31537 PMC1857251 17476347

[B52] Lin Y. Xu S. Zeng D. Ni X. Zhou M. Zeng Y. (2017). Disruption in the cecal microbiota of chickens challenged with *Clostridium perfringens* and other factors was alleviated by Bacillus licheniformis supplementation. PloS one 12, e0182426. 10.1371/journal.pone.0182426 28771569 PMC5542615

[B53] Liu S. D. Song M. H. Yun W. Lee J. H. Kim H. B. Cho J. H. (2019). Effect of carvacrol essential oils on immune response and inflammation-related genes expression in broilers challenged by lipopolysaccharide. Poult. Sci. 98, 2026–2033. 10.3382/ps/pey575 30590708

[B54] Liu C. Radebe S. M. Zhang H. Jia J. Xie S. Shi M. (2022). Effect of Bacillus coagulans on maintaining the integrity intestinal mucosal barrier in broilers. Veterinary Microbiol. 266, 109357. 10.1016/j.vetmic.2022.109357 35101712

[B55] Lopez-Castejon G. Brough D. (2011). Understanding the mechanism of IL-1β secretion. Cytokine & growth factor Rev. 22, 189–195. 10.1016/j.cytogfr.2011.10.001 22019906 PMC3714593

[B56] Lu M. Lee Y. Lillehoj H. S. (2022). Evolution of developmental and comparative immunology in poultry: the regulators and the regulated. Dev. Comp. Immunol. 138, 104525. 10.1016/j.dci.2022.104525 36058383

[B57] Lynn D. J. Lloyd A. T. O’Farrelly C. (2003). *In silico* identification of components of the Toll-like receptor (TLR) signaling pathway in clustered chicken expressed sequence tags (ESTs). Veterinary Immunol. Immunopathol. 93, 177–184. 10.1016/s0165-2427(03)00058-8 12814703

[B58] Ma D. Zhang M. Zhang K. Liu X. Han Z. Shao Y. (2013). Identification of three novel avian beta-defensins from goose and their significance in the pathogenesis of Salmonella. Mol. Immunol. 56, 521–529. 10.1016/j.molimm.2013.05.227 23911409

[B59] Mancabelli L. Milani C. Lugli G. A. Turroni F. Cocconi D. van Sinderen D. (2017). Identification of universal gut microbial biomarkers of common human intestinal diseases by meta-analysis. FEMS Microbiol. Ecol. 93, fix153. 10.1093/femsec/fix153 29126267

[B60] McDevitt R. M. Brooker J. D. Acamovic T. Sparks N. H. C. (2006). Necrotic enteritis; a continuing challenge for the poultry industry. World's Poult. Sci. J. 62, 221–247. 10.1079/wps200593

[B61] McDougald L. R. (1998). Intestinal protozoa important to poultry. Poult. Sci. 77, 1156–1158. 10.1093/ps/77.8.1156 9706082

[B62] McGuckin M. A. Lindén S. K. Sutton P. Florin T. H. (2011). Mucin dynamics and enteric pathogens. Nat. Rev. Microbiol. 9, 265–278. 10.1038/nrmicro2538 21407243

[B63] Meade K. G. Narciandi F. Cahalane S. Reiman C. Allan B. O’Farrelly C. (2009). Comparative *in vivo* infection models yield insights on early host immune response to Campylobacter in chickens. Immunogenetics 61, 101–110. 10.1007/s00251-008-0346-7 19082824

[B64] Moore R. J. (2016). Necrotic enteritis predisposing factors in broiler chickens. Avian Pathol. 45, 275–281. 10.1080/03079457.2016.1150587 26926926

[B65] Mora Z. V. D. L. Macías-Rodríguez M. E. Arratia-Quijada J. Gonzalez-Torres Y. S. Nuño K. Villarruel-López A. (2020). *Clostridium perfringens* as foodborne pathogen in broiler production: pathophysiology and potential strategies for controlling necrotic enteritis. Animals 10, 1718. 10.3390/ani10091718 32972009 PMC7552638

[B66] Mueckler M. Thorens B. (2013). The SLC2 (GLUT) family of membrane transporters. Mol. aspects Med. 34, 121–138. 10.1016/j.mam.2012.07.001 23506862 PMC4104978

[B67] Oakley B. B. Lillehoj H. S. Kogut M. H. Kim W. K. Maurer J. J. Pedroso A. (2014). The chicken gastrointestinal microbiome. FEMS Microbiol. Lett. 360, 100–112. 10.1111/1574-6968.12608 25263745

[B68] Paiva D. McElroy A. (2014). Necrotic enteritis: applications for the poultry industry. J. Appl. Poult. Res. 23, 557–566. 10.3382/japr.2013-00925

[B69] Paiva D. M. Walk C. L. McElroy A. P. (2013). Influence of dietary calcium level, calcium source, and phytase on bird performance and mineral digestibility during a natural necrotic enteritis episode. Poult. Sci. 92, 3125–3133. 10.3382/ps.2013-03298 24235221

[B70] Paiva D. Walk C. McElroy A. (2014). Dietary calcium, phosphorus, and phytase effects on bird performance, intestinal morphology, mineral digestibility, and bone ash during a natural necrotic enteritis episode. Poult. Sci. 93, 2752–2762. 10.3382/ps.2014-04148 25143591

[B71] Palm N. W. Medzhitov R. (2009). Pattern recognition receptors and control of adaptive immunity. Immunol. Rev. 227, 221–233. 10.1111/j.1600-065X.2008.00731.x 19120487

[B72] Park S. S. Lillehoj H. S. Allen P. C. Park D. W. FitzCoy S. Bautista D. A. (2008). Immunopathology and cytokine responses in broiler chickens coinfected with Eimeria maxima and *Clostridium perfringens* with the use of an animal model of necrotic enteritis. Avian Dis. 52, 14–22. 10.1637/7997-041707-Reg 18459290

[B73] Park I. Oh S. Nam H. Celi P. Lillehoj H. S. (2022). Antimicrobial activity of sophorolipids against Eimeria maxima and *Clostridium perfringens*, and their effect on growth performance and gut health in necrotic enteritis. Poult. Sci. 101, 101731. 10.1016/j.psj.2022.101731 35176703 PMC8851262

[B74] Peek H. W. (2010). Resistance to anticoccidial drugs: Alternative strategies to control coccidiosis in broilers. Utrecht University.

[B75] Perez V. G. Jacobs C. M. Barnes J. Jenkins M. C. Kuhlenschmidt M. S. Fahey G. C. Jr (2011). Effect of corn distillers dried grains with solubles and Eimeria acervulina infection on growth performance and the intestinal microbiota of young chicks. Poult. Sci. 90, 958–964. 10.3382/ps.2010-01066 21489939

[B76] Pham V. H. Abbas W. Huang J. He Q. Zhen W. Guo Y. (2022). Effect of blending encapsulated essential oils and organic acids as an antibiotic growth promoter alternative on growth performance and intestinal health in broilers with necrotic enteritis. Poult. Sci. 101, 101563. 10.1016/j.psj.2021.101563 34823183 PMC8628017

[B77] Poritz L. S. Harris L. R. Kelly A. A. Koltun W. A. (2011). Increase in the tight junction protein claudin-1 in intestinal inflammation. Dig. Dis. Sci. 56, 2802–2809. 10.1007/s10620-011-1688-9 21748286 PMC4066382

[B78] Prescott J. F. Smyth J. A. Shojadoost B. Vince A. (2016). Experimental reproduction of necrotic enteritis in chickens: A review. Avian Pathol. 45, 317–322. 10.1080/03079457.2016.1141345 26813025

[B79] Rochell S. J. Parsons C. M. Dilger R. N. (2016). Effects of Eimeria acervulina infection severity on growth performance, apparent ileal amino acid digestibility, and plasma concentrations of amino acids, carotenoids, and α1-acid glycoprotein in broilers. Poult. Sci. 95, 1573–1581. 10.3382/ps/pew035 26933234

[B80] Rothwell L. Young J. R. Zoorob R. Whittaker C. A. Hesketh P. Archer A. (2004). Cloning and characterization of chicken IL-10 and its role in the immune response to Eimeria maxima. J. Immunol. 173, 2675–2682. 10.4049/jimmunol.173.4.2675 15294985

[B81] Sakkas P. Oikeh I. Blake D. P. Nolan M. J. Bailey R. A. Oxley A. (2018). Does selection for growth rate in broilers affect their resistance and tolerance to Eimeria maxima? Veterinary Parasitol. 258, 88–98. 10.1016/j.vetpar.2018.06.014 PMC6052249 30105985

[B82] Savan R. Ravichandran S. Collins J. R. Sakai M. Young H. A. (2009). Structural conservation of interferon gamma among vertebrates. Cytokine & growth factor Rev. 20, 115–124. 10.1016/j.cytogfr.2009.02.006 19268624 PMC2755191

[B83] Sharma M. K. Liu G. White D. L. Tompkins Y. H. Kim W. K. (2022). Effects of mixed Eimeria challenge on performance, body composition, intestinal health, and expression of nutrient transporter genes of Hy-Line W-36 pullets (0-6 wks of age). Poult. Sci. 101, 102083. 10.1016/j.psj.2022.102083 36130447 PMC9489515

[B84] Shojadoost B. Vince A. R. Prescott J. F. (2012). The successful experimental induction of necrotic enteritis in chickens by *Clostridium perfringens*: A critical review. Veterinary Res. 43, 74–12. 10.1186/1297-9716-43-74 PMC3546943 23101966

[B85] Shojadoost B. Alizadeh M. Boodhoo N. Astill J. Karimi S. H. Shoja Doost J. (2022). Effects of treatment with lactobacilli on necrotic enteritis in broiler chickens. Probiotics Antimicrob. Proteins 14, 1110–1129. 10.1007/s12602-021-09901-5 35044636

[B86] Short F. J. Gorton P. Wiseman J. Boorman K. N. (1996). Determination of titanium dioxide added as an inert marker in chicken digestibility studies. Animal feed Sci. Technol. 59, 215–221. 10.1016/0377-8401(95)00916-7

[B87] Smith D. E. Clémençon B. Hediger M. A. (2013). Proton-coupled oligopeptide transporter family SLC15: physiological, pharmacological and pathological implications. Mol. aspects Med. 34, 323–336. 10.1016/j.mam.2012.11.003 23506874 PMC3602806

[B88] Songer J. G. Meer R. R. (1996). Genotyping ofClostridium perfringensby polymerase chain reaction is a useful adjunct to diagnosis of clostridial enteric disease in animals. Anaerobe 2, 197–203. 10.1006/anae.1996.0027

[B89] Speier J. S. Yadgary L. Uni Z. Wong E. A. (2012). Gene expression of nutrient transporters and digestive enzymes in the yolk sac membrane and small intestine of the developing embryonic chick. Poult. Sci. 91, 1941–1949. 10.3382/ps.2011-02092 22802189

[B90] Sperandio B. Regnault B. Guo J. Zhang Z. Stanley S. L. Jr Sansonetti P. J. (2008). Virulent Shigella flexneri subverts the host innate immune response through manipulation of antimicrobial peptide gene expression. J. Exp. Med. 205, 1121–1132. 10.1084/jem.20071698 18426984 PMC2373844

[B91] Stanley D. Wu S. B. Rodgers N. Swick R. A. Moore R. J. (2014). Differential responses of cecal microbiota to fishmeal, Eimeria and *Clostridium perfringens* in a necrotic enteritis challenge model in chickens. PloS one 9, e104739. 10.1371/journal.pone.0104739 25167074 PMC4148237

[B92] Su S. Miska K. B. Fetterer R. H. Jenkins M. C. Wong E. A. (2014). Expression of digestive enzymes and nutrient transporters in Eimeria acervulina-challenged layers and broilers. Poult. Sci. 93, 1217–1226. 10.3382/ps.2013-03807 24795315

[B93] Sumners L. H. Miska K. B. Jenkins M. C. Fetterer R. H. Cox C. M. Kim S. (2011). Expression of Toll-like receptors and antimicrobial peptides during Eimeria praecox infection in chickens. Exp. Parasitol. 127, 714–718. 10.1016/j.exppara.2010.12.002 21176773

[B94] Susta L. Diel D. G. Courtney S. Cardenas-Garcia S. Sundick R. S. Miller P. J. (2015). Expression of chicken interleukin-2 by a highly virulent strain of Newcastle disease virus leads to decreased systemic viral load but does not significantly affect mortality in chickens. Virology J. 12, 1–17. 10.1186/s12985-015-0353-x 26253150 PMC4528788

[B95] Takehara M. Seike S. Sonobe Y. Bandou H. Yokoyama S. Takagishi T. (2019). *Clostridium perfringens* α-toxin impairs granulocyte colony-stimulating factor receptor-mediated granulocyte production while triggering septic shock. Commun. Biol. 2, 45. 10.1038/s42003-019-0280-2 30729183 PMC6355902

[B96] Takeuchi O. Akira S. (2010). Pattern recognition receptors and inflammation. Cell. 140, 805–820. 10.1016/j.cell.2010.01.022 20303872

[B97] Tau G. Rothman P. (1999). Biologic functions of the IFN‐γ receptors. Allergy 54, 1233–1251. 10.1034/j.1398-9995.1999.00099.x 10688427 PMC4154595

[B98] Teng P. Y. Yadav S. de Souza Castro F. L. Tompkins Y. H. Fuller A. L. Kim W. K. (2020). Graded Eimeria challenge linearly regulated growth performance, dynamic change of gastrointestinal permeability, apparent ileal digestibility, intestinal morphology, and tight junctions of broiler chickens. Poult. Sci. 99, 4203–4216. 10.1016/j.psj.2020.04.031 32867964 PMC7598010

[B99] Teng P. Y. Yadav S. Shi H. Kim W. K. (2021). Evaluating endogenous loss and standard ileal digestibility of amino acids in response to the graded severity levels of E. maxima infection. Poult. Sci. 100, 101426. 10.1016/j.psj.2021.101426 34547620 PMC8463777

[B100] Tian X. Shao Y. Wang Z. Guo Y. (2016). Effects of dietary yeast β-glucans supplementation on growth performance, gut morphology, intestinal *Clostridium perfringens* population and immune response of broiler chickens challenged with necrotic enteritis. Animal Feed Sci. Technol. 215, 144–155. 10.1016/j.anifeedsci.2016.03.009

[B101] Townes C. L. Michailidis G. Nile C. J. Hall J. (2004). Induction of cationic chicken liver-expressed antimicrobial peptide 2 in response to *Salmonella enterica* infection. Infect. Immun. 72, 6987–6993. 10.1128/IAI.72.12.6987-6993.2004 15557621 PMC529109

[B102] Tsiouris V. Georgopoulou I. Batzios C. Pappaioannou N. Ducatelle R. Fortomaris P. (2015a). High stocking density as a predisposing factor for necrotic enteritis in broiler chicks. Avian Pathol. 44, 59–66. 10.1080/03079457.2014.1000820 25563065

[B103] Tsiouris V. Georgopoulou I. Batzios C. Pappaioannou N. Ducatelle R. Fortomaris P. (2015b). The effect of cold stress on the pathogenesis of necrotic enteritis in broiler chicks. Avian Pathol. 44, 430–435. 10.1080/03079457.2015.1083094 26642742

[B104] Ulluwishewa D. Anderson R. C. McNabb W. C. Moughan P. J. Wells J. M. Roy N. C. (2011). Regulation of tight junction permeability by intestinal bacteria and dietary components. J. Nutr. 141, 769–776. 10.3945/jn.110.135657 21430248

[B105] van Dijk A. Veldhuizen E. J. Haagsman H. P. (2008). Avian defensins. Veterinary Immunol. Immunopathol. 124, 1–18. 10.1016/j.vetimm.2007.12.006 PMC7112556 18313763

[B106] Van Dijk A. Molhoek E. M. Bikker F. J. Yu P. L. Veldhuizen E. J. A. Haagsman H. P. (2011). Avian cathelicidins: paradigms for the development of anti-infectives. Veterinary Microbiol. 153, 27–36. 10.1016/j.vetmic.2011.03.028 21530107

[B107] Van Immerseel F. Buck J. D. Pasmans F. Huyghebaert G. Haesebrouck F. Ducatelle R. (2004). *Clostridium perfringens* in poultry: an emerging threat for animal and public health. Avian Pathol. 33, 537–549. 10.1080/03079450400013162 15763720

[B108] Van Immerseel F. Rood J. I. Moore R. J. Titball R. W. (2009). Rethinking our understanding of the pathogenesis of necrotic enteritis in chickens. Trends Microbiol. 17, 32–36. 10.1016/j.tim.2008.09.005 18977143

[B109] Wade B. Keyburn A. (2015). The true cost of necrotic enteritis. World Poult. 31, 16–17.

[B110] Wade B. Keyburn A. L. Seemann T. Rood J. I. Moore R. J. (2015). Binding of *Clostridium perfringens* to collagen correlates with the ability to cause necrotic enteritis in chickens. Veterinary Microbiol. 180, 299–303. 10.1016/j.vetmic.2015.09.019 26455806

[B111] Walker R. A. Sharman P. A. Miller C. M. Lippuner C. Okoniewski M. Eichenberger R. M. (2015). RNA Seq analysis of the Eimeria tenella gametocyte transcriptome reveals clues about the molecular basis for sexual reproduction and oocyst biogenesis. BMC genomics 16, 94–20. 10.1186/s12864-015-1298-6 25765081 PMC4345034

[B112] Wang H. Ni X. Qing X. Liu L. Lai J. Khalique A. (2017). Probiotic enhanced intestinal immunity in broilers against subclinical necrotic enteritis. Front. Immunol. 8, 1592. 10.3389/fimmu.2017.01592 29209325 PMC5701917

[B113] Wang H. Latorre J. D. Bansal M. Abraha M. Al-Rubaye B. Tellez-Isaias G. (2019). Microbial metabolite deoxycholic acid controls Clostridium perfringens-induced chicken necrotic enteritis through attenuating inflammatory cyclooxygenase signaling. Sci. Rep. 9, 14541. 10.1038/s41598-019-51104-0 31601882 PMC6787040

[B114] Wei X. Yang Z. Rey F. E. Ridaura V. K. Davidson N. O. Gordon J. I. (2012). Fatty acid synthase modulates intestinal barrier function through palmitoylation of mucin 2. Cell. host microbe 11, 140–152. 10.1016/j.chom.2011.12.006 22341463 PMC3285413

[B115] Whelan R. A. Doranalli K. Rinttilä T. Vienola K. Jurgens G. Apajalahti J. (2019). The impact of Bacillus subtilis DSM 32315 on the pathology, performance, and intestinal microbiome of broiler chickens in a necrotic enteritis challenge. Poult. Sci. 98, 3450–3463. 10.3382/ps/pey500 30452717 PMC6698186

[B116] Williams R. B. Marshall R. N. La Ragione R. M. Catchpole J. (2003). A new method for the experimental production of necrotic enteritis and its use for studies on the relationships between necrotic enteritis, coccidiosis and anticoccidial vaccination of chickens. Parasitol. Res. 90, 19–26. 10.1007/s00436-002-0803-4 12743800

[B117] Williams R. B. (2001). Quantification of the crowding effect during infections with the seven eimeria species of the domesticated fowl: its importance for experimental designs and the production of oocyst stocks. Int. J. Parasitol. 31, 1056–1069. 10.1016/s0020-7519(01)00235-1 11429169

[B118] Williams R. B. (2005). Intercurrent coccidiosis and necrotic enteritis of chickens: rational, integrated disease management by maintenance of gut integrity. Avian Pathol. 34, 159–180. 10.1080/03079450500112195 16191699

[B119] Wu S. B. Rodgers N. Choct M. (2010). Optimized necrotic enteritis model producing clinical and subclinical infection of *Clostridium perfringens* in broiler chickens. Avian Dis. 54, 1058–1065. 10.1637/9338-032910-Reg.1 20945788

[B120] Wu Y. Shao Y. Song B. Zhen W. Wang Z. Guo Y. (2018). Effects of Bacillus coagulans supplementation on the growth performance and gut health of broiler chickens with Clostridium perfringens-induced necrotic enteritis. J. animal Sci. Biotechnol. 9, 9–14. 10.1186/s40104-017-0220-2 PMC5784659 29416856

[B121] Yang Q. Chen B. Robinson K. Belem T. Lyu W. Deng Z. (2022). Butyrate in combination with forskolin alleviates necrotic enteritis, increases feed efficiency, and improves carcass composition of broilers. J. Animal Sci. Biotechnol. 13, 3. 10.1186/s40104-021-00663-2 PMC8830124 35139922

[B122] Zanu H. K. Kheravii S. K. Morgan N. K. Bedford M. R. Swick R. A. (2020). Interactive effect of 2 dietary calcium and phytase levels on broilers challenged with subclinical necrotic enteritis: part 1—broiler performance, gut lesions and pH, bacterial counts, and apparent ileal digestibility. Poult. Sci. 99, 4861–4873. 10.1016/j.psj.2020.05.033 32988523 PMC7810914

[B123] Zhang B. Lv Z. Li Z. Wang W. Li G. Guo Y. (2018). Dietary L-arginine supplementation alleviates the intestinal injury and modulates the gut microbiota in broiler chickens challenged by *Clostridium perfringens* . Front. Microbiol. 9, 1716. 10.3389/fmicb.2018.01716 30108569 PMC6080643

